# Supplementary material for: Experiences and perceptions of trial participants and healthcare professionals in the UK Frozen Shoulder Trial (UK FROST): a nested qualitative study
Source: BMJ Open. 2021 Jun 11;11(6):e040829. doi: 10.1136/bmjopen-2020-040829 (PMC8202103; doi:10.1136/bmjopen-2020-040829)
Supplement: Author's manuscript [file bmjopen-2020-040829.draft_revisions.pdf]

BMJ Open

BMJ Open is committed to open peer review. As part of this commitment we make the peer review history of every article we publish publicly available.

When an article is published we post the peer reviewers' comments and the authors' responses online. We also post the versions of the paper that were used during peer review. These are the versions that the peer review comments apply to.

The versions of the paper that follow are the versions that were submitted during the peer review process. They are not the versions of record or the final published versions. They should not be cited or distributed as the published version of this manuscript.

BMJ Open is an open access journal and the full, final, typeset and author-corrected version of record of the manuscript is available on our site with no access controls, subscription charges or pay-per-view fees (http://bmjopen.bmj.com).

If you have any questions on BMJ Open's open peer review process please email info.bmjopen@bmj.com

The experiences of patients and healthcare professionals in the United Kingdom Frozen Shoulder Trial (UK FROST): A nested qualitative study.

Journal:	*BMJ Open*
Manuscript ID	bmjopen-2020-040829
Article Type:	Original research
Date Submitted by the Author:	25-May-2020
Complete List of Authors:	Srikesavan, Cynthia; University of Oxford, Nuffield Department of Orthopaedics, Rheumatology, and Musculoskeletal Sciences<br>Toye, Francine; Oxford University Hospitals NHS Foundation Trust, Nuffield Orthopaedic Centre, Physiotherapy Research Unit<br>Brealey, Stephen; University of York, York Trials Unit, ARRC Building, Department of Health Sciences.<br>Goodchild, Lorna; The Physiotherapy Practice<br>Northgraves, Matthew; Hull York Medical School<br>Charalambous, C; Blackpool Teaching Hospitals NHS Foundation Trust; University of Central Lancashire, School of Medicine<br>Rangan, Amar; University of York, York Trials Unit, ARRC Building, Department of Health Sciences<br>Lamb, Sarah; University of Exeter, College of Medicine and Health; University of Oxford, Nuffield Department of Orthopaedics, Rheumatology, and Musculoskeletal Sciences
Keywords:	Elbow & shoulder < ORTHOPAEDIC & TRAUMA SURGERY, Musculoskeletal disorders < ORTHOPAEDIC & TRAUMA SURGERY, Shoulder < ORTHOPAEDIC & TRAUMA SURGERY

SCHOLARONE™
Manuscripts

I, the Submitting Author has the right to grant and does grant on behalf of all authors of the Work (as defined in the below author licence), an exclusive licence and/or a non-exclusive licence for contributions from authors who are: i) UK Crown employees; ii) where BMJ has agreed a CC-BY licence shall apply, and/or iii) in accordance with the terms applicable for US Federal Government officers or employees acting as part of their official duties; on a worldwide, perpetual, irrevocable, royalty-free basis to BMJ Publishing Group Ltd ("BMJ") its licensees and where the relevant Journal is co-owned by BMJ to the co-owners of the Journal, to publish the Work in this journal and any other BMJ products and to exploit all rights, as set out in our licence.

The Submitting Author accepts and understands that any supply made under these terms is made by BMJ to the Submitting Author unless you are acting as an employee on behalf of your employer or a postgraduate student of an affiliated institution which is paying any applicable article publishing charge ("APC") for Open Access articles. Where the Submitting Author wishes to make the Work available on an Open Access basis (and intends to pay the relevant APC), the terms of reuse of such Open Access shall be governed by a Creative Commons licence – details of these licences and which Creative Commons licence will apply to this Work are set out in our licence referred to above.

Other than as permitted in any relevant BMJ Author's Self Archiving Policies, I confirm this Work has not been accepted for publication elsewhere, is not being considered for publication elsewhere and does not duplicate material already published. I confirm all authors consent to publication of this Work and authorise the granting of this licence.

For peer review only

Title page

Title of the article

The experiences of patients and healthcare professionals in the United Kingdom Frozen Shoulder Trial (UK FROST): A nested qualitative study.

Full name, postal address and e-mail of the corresponding author

Dr Francine Toye (Ph.D, M.A Archaeology & Anthropology Cantab)
Qualitative Research Lead, Physiotherapy Research Unit,
Nuffield Orthopaedic Centre,
Oxford University Hospitals NHS Foundation Trust,
Windmill Road, Oxford OX3 7HE.
United Kingdom.

Email: francine.toye@ouh.nhs.uk.

Full name, department, institution, city and country of all co-authors

[1]Cynthia Srikesavan, [2]Francine Toye, [3]Stephen Brealey, [4]Lorna Goodchild, [5]Matthew Northgraves, [6]Charalambos P. Charalambous, [7]Amar Rangan, [8]Sarah E Lamb.

Affiliations

[1]Nuffield Department of Orthopaedics, Rheumatology, and Musculoskeletal Sciences, University of Oxford, Windmill road, Oxford, United Kingdom.
[2]Nuffield Orthopaedic Centre, Oxford University Hospitals NHS Foundation Trust, Windmill Road, Oxford, United Kingdom.
[3, 7]York Trials Unit, ARRC building, Department of Health Sciences, University of York, ARRC Building, York, United Kingdom.
[4]The Physiotherapy Practice, South Shields, United Kingdom.
[5]Hull York Medical School, Hull, United Kingdom.
[6]Blackpool Teaching Hospitals NHS Trust, Blackpool, and School of Medicine, University of Central Lancashire, Preston, United Kingdom.
[8]College of Medicine and Health, University of Exeter & Nuffield Department of Orthopaedics, Rheumatology, and Musculoskeletal Sciences, University of Oxford, Windmill road, Oxford, United Kingdom.

Word count, excluding title page, abstract, references, figures and tables: 4697

Keywords: Adhesive capsulitis; Arthroscopic capsular release; Frozen shoulder; Interviews; Manipulation under anaesthesia; Physiotherapy.

Abstract

Abstract word count: 296

Objectives

To explore the experiences of patients and healthcare professionals in the United Kingdom Frozen Shoulder Trial (UK FROST), a multi-centre randomised controlled trial that compared manipulation under anaesthesia (MUA), arthroscopic capsular release (ACR) with a 12-week early structured physiotherapy programme (ESP) in people with unilateral frozen shoulder referred to secondary care.

Design

Nested qualitative study with semi-structured interviews.

Setting

Face-to-face or telephone interviews with trial participants, surgeons and physiotherapists.

Participants

44 participants (ESP: 14; MUA: 15; ACR: 15), and 8 surgeons and 8 physiotherapists who delivered the treatments in the trial.

Results

Participants described that frozen shoulder had a major impact on all aspects of their life. They found UK FROST treatments acceptable and satisfactory in terms of content, delivery and treatment benefits. Participants in all arms experienced improvements in pain, shoulder movements, and function. Participants said they would choose the same treatment that they received in the trial.

Surgeons and physiotherapists used a stage-based treatment approach in routine practice, depending on the severity and duration of symptoms. They had mixed feelings about the effectiveness of UK FROST treatments and felt diabetes would lead to poorer outcomes. Physiotherapists had concerns about the capacity to deliver the number of UK FROST physiotherapy sessions in routine clinical settings. Surgeons and physiotherapists stressed the value of including hydro-dilatation as a comparator of other treatment options.

Common perceptions of participants, surgeons and physiotherapists were: 1) Pain relief and return of shoulder movements and function are important outcomes; 2) Steroids help in pain relief and to initiate exercises; 3) Participants had treatment preferences when entering the trial and; 4) Adherence to exercises leads to better outcomes.

Conclusion

Our findings provide important insights to further guide treatment decision-making in frozen shoulder and in the design of future trials evaluating this condition.

Keywords: Adhesive capsulitis; Arthroscopic capsular release; Frozen shoulder; Interviews; Manipulation under anaesthesia; Physiotherapy.

For peer review only

ARTICLE SUMMARY

Strengths and limitations of this study

- Interviews were conducted by a researcher who was not involved in UK FROST and using open-ended questions.
- Interview codes and themes were reviewed by an experienced researcher to ensure rigour of analysis and interpretation of data.
- Interviews were conducted with patients and healthcare professionals who participated in UK FROST and therefore the findings may not be transferable outside this context.
- 93% of the interviews were telephone-based. It is uncertain if interviewees would have expressed differently in face-to-face interviews.
- Those who did not participate in the interviews may have had different experiences.

For peer review only

Main text word count: 4697

INTRODUCTION

Frozen shoulder or adhesive capsulitis is a chronic shoulder condition, commonly affecting people aged between 50 and 60 years [1]. Women are affected four times than men [2], while the prevalence rate is around 20% in people with diabetes [2]. Frozen shoulder is characterised by three clinical stages that often overlap [3, 4]: 1) a painful stage (3 to 8 months), 2) a stiffness (frozen) stage (4 to 12 months) with progressive loss of active and passive shoulder movements and 3) a thawing stage (12 to 42 months) with gradual recovery in most people [2].

Frozen shoulder is managed by non-surgical or surgical treatments [3, 5-8]. However, there is no high-quality evidence to recommend one treatment over another [3, 7]. There is also very limited literature on patient experience of frozen shoulder [9, 10]. The United Kingdom Frozen Shoulder Trial (UK FROST) is a multi-centre, pragmatic randomised controlled trial in adults with frozen shoulder [11]. It evaluated the clinical and cost-effectiveness of two common surgical treatments: manipulation under anaesthesia (MUA) and arthroscopic capsular release (ACR) both followed by up to 12 weeks of post-procedural physiotherapy (PPP) with a 12-week early structured physiotherapy (ESP) programme that included an intra-articular steroid injection. Detailed descriptions of the trial treatments, including the specifically developed ESP and PPP pathways for the trial are available [11].

A nested qualitative study was conducted to explore the experience and acceptability of the treatments to participants and healthcare professionals (surgeons and physiotherapists) who delivered the treatments.

METHODS

Ethics approval was obtained from the NRES Committee North East -Newcastle and North Tyneside 2 ethics committee (14/NE/1176) and Health Research Authority (HRA).

Participants who took part in the trial had a clinical diagnosis of unilateral frozen shoulder and were randomly assigned to MUA, ACR or ESP treatment arms in the ratio of 2:2:1. Those who had agreed to be contacted by the trial team were invited for interviews at approximately 12 months post-randomisation, which coincided with the primary endpoint of the trial [11]. As gender and diabetes are likely to have an impact on the treatment outcomes [2, 11], we proposed to include an equal number of men and women, and those with and without diabetes. We planned to interview up to 45 participants and 15 surgeons and physiotherapists [11].

After obtaining written consent, one of the authors (CS) trained in qualitative research methods and not involved in the delivery of UK FROST treatments and outcome measurements conducted the interviews. Semi-structured interview guides with open-ended questions were used.. Given the geographical spread of those participated, interviews were conducted either face-to-face or via telephone. Interviews were audio-recorded using a digital recorder, transcribed verbatim, and anonymised.

A constructivist approach, which takes the stance that findings are co-constructed by researcher and interviewees [12], was used. The interviews were analysed using constant comparative method [12, 13] to develop categories and themes with a shared essence. The QSR International's NVivo 11

For peer review only - http://bmjopen.bmj.com/site/about/guidelines.xhtml

software [14] was used to organise and assist analysis of the data. All interviews were analysed by CS. To enhance credibility of the findings, categories and themes were reviewed by an experienced qualitative researcher (FT). The standards for reporting qualitative research (SRQR) guidelines were used to report the study [15].

Patient and public involvement

The patient interview guide was developed following discussions with patients with frozen shoulder, research team, a physiotherapist and surgeon with expertise in this area, and a literature review.

RESULTS

Sixty interviews (Participants: 44; Surgeons: 8 and Physiotherapists: 8) were completed between August 2016 and January 2018. Most interviews (56/60, 93%) were conducted via telephone. Four interviews were held at the clinic offices of surgeons and physiotherapists. Though constant comparison was used to analyse data, a formal theory was not developed [13]. The analysis was stopped when useful themes emerged.

A. Interviews with trial participants

A flow diagram showing the number of patients invited, excluded and participated in the interviews is presented in Figure 1. Participant characteristics are presented in Table 1. Apart from two participants, all other participants received their allocated treatment.

Five themes were identified. The themes were not different between men and women; and between those with and without diabetes.

Table 1: Characteristics of the participants interviewed

UK FROST arms	Male	Female	Age<br>Median (IQR) in years	Diabetic	Non-diabetic
ESP	5	9	58 (51 to 63.5)	3	11
MUA	8	7	55 (53 to 57.5)	5	10
ACR	7	8	59 (53 to 69)	5	10

UK FROST: United Kingdom Frozen Shoulder Trial; IQR: Inter-quartile range; ESP: Early Structured Physiotherapy; MUA: Manipulation Under Anaesthesia; ACR: Arthroscopic Capsular Release.

For peer review only

1) Living with frozen shoulder

Frozen shoulder had a negative impact on all areas of life. Participants described it as a a painful, restrictive and disabling condition, attributing it to a historic shoulder injury or tasks such as gardening and lifting heavy weights. At the onset, there was either a sudden sharp pain or a constant dull ache that progressed over time. During the course of frozen shoulder, participants had pain, restricted shoulder movements; sleep disturbances, limitations in day-to-day activities, and reduced ability to participate in leisure, work, and social activities. Supplementary file 1 presents participants' narratives of the problems experienced due to frozen shoulder.

Participants described seeking medical help when they realised the condition was beginning to interfere with their daily activites. Delays were experienced in getting a quick diagnosis and specialist referrals in the National Health Service (NHS). In retrospect, participants emphasised the need to seek early medical help and the need for a quicker NHS pathways in terms of diagnosis and referrals. They were concerned with their upper limb disability from frozen shoulder and were keen on getting it sorted in UK FROST.

> *"I would say go straight to your doctor and get them to refer you. That would be the first thing because it doesn't just seem to go away of its own accord which I possibly thought initially and that's why I delayed in going to the doctor's in the first place".* (ESP)

> *"Just speed it up. It was, I think I went to see the GP at the beginning of November, and it wasn't really till the following January before I got any kind of treatment other than pain relief, by which time I'd lost all movement".* (MUA)

2) Acceptability of treatments –Satisfaction and improved outcomes

Except for two participants in the ESP arm, all other participants were satisfied with the UK FROST treatments they received. Pain relief and return of shoulder movements and function were considered as important treatment outcomes. Participants in all arms experienced improvements in pain, movements and function.

Pain:
There was a substantial reduction in pain in all treatment arms. Some participants reported ongoing occasional pain during certain end-range activities: however, this did not impact their daily functioning.

> *"When I went to the surgeon I was injected into my shoulder and the pain down my arm that more or less went straightaway."* (ESP)

> *"So at the beginning I said the pain was ten and now after all my physios, I'd say it was, I'd say it was about two now."* (MUA)

> *"I mean the pain in the beginning was just horrendous, it was really, really sore, really painful but after I'd had the physiotherapy, it was… I've got no pain at all now."* (ACR)

Movements:
Improvement in movements was seen across all the treatment arms.

For peer review only

"I could tell initially straightaway that my movement was starting to come, within a few days I could tell a difference of doing the exercises and as the weeks went on, it was just got better and better and by the time the twelve weeks was up, I virtually had full movements with no pain or anything, it was brilliant!" (ESP)

"After a few days I was doing my exercises and I was quite surprised already how much movement I had back and then it was regular physio appointments up at the hospital just to keep moving things around and that went really well…the physiotherapy was actually really, really beneficial." (MUA)

In the ACR arm, improvement in movements was thought to be quicker than participants had expected.

"I felt that the physiotherapy I received was marvellous and improved the range of movements or showed me how to keep that range of movements much quicker than they did on the right-hand side, so I felt that everything went along fine, and I've got no complaints at all, none." (ACR)

"*It is almost like you have had a quick fix to fix your shoulder then you move on and I think personally for me because the surgery went very well and almost after a couple of weeks I was back to normal.*" (ACR)

Function:
Improvement in function allowed participants to perform activities of daily living.

"I can lift my arm above my head now, you know? I can carry stuff, and I can lift it above my waist, and I can actually go swimming, you know? I can swim now." (ESP)

"My little everyday things have come back; I have come back, yes". (MUA)

"I can do everything – there's nothing that I can't do; I can wash my back, I can put my bra on, fasten it at the back, I can fasten my skirt at the side and the back now, there's nothing I can't do before I had the frozen shoulder everything I could do then I can now do again". (ACR)

3) Adherence to exercises

Participants described that ESP and PPP were difficult to begin with, but became easier on subsequent sessions.

"It (Physiotherapy) *was agony but from every session there was slight improvement, so for every kind, every session I had, I had more movement and less pain, so it got to a point where some days I would go down and I had no pain at all."* (ESP)

However, participants were aware of the benefits of exercises and persevered with their home exercises during the study period.

For peer review only

"It was uncomfortable but I tried really hard with them to be honest because it was so awful when I couldn't use my arm that I knew that the only way to help it was to do them as much as I could." (MUA)

Several participants said they did not continue their home exercises after the trial because they felt they had regained adequate shoulder function. Instead, they maintained their shoulder mobility by being functionally active.

"*I'm working with my shoulder all the time so I'm not doing the exercises that the hospital gave me, because I'm working my, I'm swimming, I'm doing…I go on long walks, I take the dog out and what have you, so I'm using my arm*." (ESP)

4) Personal treatment preferences

Participants had mixed treatment preferences before the trial. Some considered physiotherapy to be ineffective, while few wanted to avoid the risks of surgery. Some preferred the less invasive MUA while some thought of ACR as the final solution. Some didn't have any preference at all.

"I'm not really too sure why I wouldn't choose physiotherapy. I just think surgery seems a more final option. Physiotherapy, it might work, it may help, it may not. But to me, if I was given surgery, the surgery would work. I had more faith in the surgery working than the physiotherapy itself." (ACR)

"Well I didn't want to go to surgery or anything like that, so I just had the needle in my shoulder. I don't think I'd have wanted to go surgery at my age" (ESP)

Despite preferences at the outset, at the end of the trial, there was a sense that participants would choose the same treatment they received in the UK FROST. In retrospect, some participants in ESP and MUA said that they would choose surgery as a permanent and time-saving solution for their shoulder problem.

"Oh no I would have the same treatment. As I say I was only there for ten weeks and I mean, on my eleventh week I was still hell of a lot better." (ESP)

5) Participation in UK FROST

Participants had positive experiences in participating in UK FROST. They described altruistic and personal reasons for taking part in the trial. There was an overall sense that the trial questionnaires were relevant, simple and straightforward to complete.

"Because I'm all in favour, if you can do something to help other people not go through the misery that you've been through, and gone through, then I would do it". (ESP)

Participants felt that their treatment packages were well coordinated and did not require any modification. The surgical procedures were clearly explained and physiotherapists who delivered ESP and PPP were supportive and helpful. Participants also liked to see the same physiotherapist for developing good connections and rapport throughout the study period. Some ESP and ACR arm participants felt that the exercises time consuming and were difficult to fit into their daily life. Some

For peer review only

said that they would have liked more information about continuing exercises after the trial and exercising when in pain.

> *"Operation, I believe obviously went fantastic, went really well. It was just a case of waking up, recovery*."(ACR)

> *"Well personally I found it quite difficult to make sure I had them that many times in the day. I'm not too sure how somebody working or having a family could actually manage to fit it in because as I say, by the time going towards the middle to the end of the programme…"* (ESP)

B. Interviews with surgeons and physiotherapists

A flow diagram showing the number of health professionals invited, excluded and participated in the interviews is presented in Figure 2. The characteristics of surgeons and physiotherapists are presented in Table 2.

Four themes were identified.

Table 2: Characteristics of the surgeons and physiotherapists interviewed

	**Gender**		**Years of experience in treating shoulder conditions**
	Male	Female	Median (IQR) years
Surgeons	7	1	11 (7 to16.25)
Physiotherapists	--	8	13.5 (12 to 15.75)

IQR: Inter-quartile range

1) A stage-based approach in routine clinical practice

In routine practice, surgeons and physiotherapists treated patients according to the clinical symptoms of the particular stage of frozen shoulder. During the early painful stage, pain control by steroid injections was prioritised to allow gradual progression of exercises.

> Physiotherapist 1: "*I would always tend to go down the glenohumeral joint injection first to see if that settled things down and to give me a window where I could then push them with regards to their exercises."*

> Surgeon 6: "*A humeral steroid injection is beneficial for pain relief. It allows them to do the physiotherapy. So, I do use that a lot"*

Patient education was the core treatment component prescribed by the physiotherapists.

> Physiotherapist 5: "*And then it's education; tell them all about the condition, what we know about the condition, let them know that it is going to get better over time, and it may not resolve fully and so on. So we'll basically educate them, reassure them.*"

Surgeons felt that exercises alone might aggravate pain during the early painful stage.

> Surgeon 2: "*Physiotherapy in isolation without an injection I've found most trial participants present having had really pain that's not manageable because they're trying to stretch and rehab a painful shoulder from frozen shoulder just increases their pain*".

During the stiffness-dominant or post-surgical stages, physiotherapists focused on improving shoulder movements, strength, and function through intensive exercise programmes.

> Physiotherapist 4: "[if] *I know that pain is not a problem then I can push them, I can do the more vigorous stretches, I can manipulate their joints or mobilise their joints and I put them on a more gym-based or exercise-based programme…I will go and more push them towards reaching their more functional goals and more towards achieving their return to work...*".

Surgery was considered the final treatment option for people with severe or long-standing symptoms and those resistant to conservative interventions (e.g. people with diabetes).

> Physiotherapist 2: "*Generally, the people that don't respond to physio often will end up seeing a surgeon at some point. So the tricky more longer-term frozen shoulder trial participants are the ones with diabetes that tend to have more problems and end up requiring treatment.*"

> Surgeon 6: "*So, if a shoulder is extremely stiff, tight and quite painful I would probably avoid doing a manipulation on these trial participants and my preference would be ACR if the longevity of the stiffness and the symptoms is quite prolonged again my preference would be an ACR.*"

Surgeons chose ACR in relation to faster recovery, low risk of humeral fractures, and familiarity with the procedure. Surgeons also considered that exercises after surgery were important for recovery.

> Surgeon 1: *"I wasn't really doing manipulations because of my experience where a fracture had been caused. So, generally speaking, I was doing ACR. "*

> *Surgeon 3: "We do believe in physiotherapy afterwards. One is to have specialised physiotherapists who can allay their fears and talk about their fitness, talk about their recovery, talk about the timescales and the pain".*

2) Expectations and preferences about UK FROST treatments

Although health professionals said that they maintained equipoise, some described mixed expectations regarding UK FROST treatments. For example, some felt that ESP would not be as effective as surgery.

For peer review only

Surgeon 7: "*My expectation is that physio won't work. My expectation is that the other two are probably Equivocal."*

Some felt that ACR would perform better than ESP, whilst others felt that MUA would be comparable to ACR. There were also those who expected similar outcomes across arms.

Physiotherapist 6*: "I'm expecting the MUAs to be surprisingly better than I would expect. I think the arthrolysis do great anyway and the physio is an unfair one."*

Physiotherapists felt that the UK FROST physiotherapy programmes and the exercise booklet gave flexibility in choosing exercises that were compatible with their routine practice. There were a few suggestions made, for example, structuring the treatment components to suit the stages of frozen shoulder, and spreading out the 12 weekly sessions over 6 months and conducting group sessions.

Physiotherapist 2: "I *think the interventions that were on the booklet were what I would use generally. There was always an option there for me to tick off what I would do so I was in agreement with the options that were there and in agreement with the options that they actually, didn't want you to use*".

Physiotherapist 5: "*I think group sessions would be really useful because patients get a lot from each other, and having experience group sessions with other clients with different pathologies, you know, they find that really reassuring…*"

Physiotherapists also commented on the feasibility of delivering the UK FROST physiotherapy programmes within the NHS. There was a sense that it would be difficult to deliver the number of UK FROST physiotherapy sessions in routine practice.

Physiotherapist 2: "*We don't normally get the luxury of being able to see patients as often as the FROST Trial was letting us. I think it was 12 treatments we could have overall."*

Physiotherapist 4: "*… they* (Trial participants) *were seen with the 24 hours' post-surgery and they had twelve sessions which is a luxury because in our Trust, that is never, not going to happen and that never used to happen".*

Physiotherapist 7: *"In general practice, well if somebody is improving and they're self-managing, then we don't need to be seeing these patients every week and our service would not allow us to be able to see them every week".*

Surgeons and physiotherapists suggested hydro-dilatation would be an easy to use, less invasive and inexpensive treatment and an appropriate alternative to combat NHS surgery waiting lists. They felt hydro-dilatation should have been included as a potential comparator in UK FROST.

Surgeon 5: "*I'd definitely have a hydro-dilatation group because part of your trial is trying to work out if the cheaper operation is better than the more expensive operation and hydro-dilatations probably gained quite popularity since we started the trial design and it reflects current practice*."

For peer review only

Physiotherapist 6: "*It needs hydro-dilatation in it. I personally think it gets really good results on a big bulk of trial participants and it's a wasted opportunity to have done this study and not have that as one of the arms".*

3) Factors that influence treatment outcomes

Similar to trial participants, surgeons and physiotherapists also felt that reduced pain, improved movement and function were important treatment outcomes. They considered that patient engagement and positive treatment expectations would contribute to better outcomes, whilst diabetes would have a negative impact on prognosis.

Physiotherapist 5: "*I think the expectations and belief is probably the most noticeable factor that affects people's outcome; if they believe something is the right thing, the best thing for them, they seem to do well*."

Surgeon 8: *"...I've seen plenty of them of male diabetics that say, "Well, I've been stiff for three years," or two and a half years, that's not uncommon"*

4) Participants' experience in the trial

Surgeons and physiotherapists described that some patients declined to take part in UK FROST because they had previous physiotherapy that did not work and hence wanted to avoid randomisation into the ESP.

Surgeon 8: "*Many of them they say, "Look, I would love to contribute to the greater good and be involved in clinical trials, but I've come to the point that I will not consent for physiotherapy if I was randomised to that...*"

They also described that participants came with fixed ideas of what treatment they wanted in the trial.

Physiotherapist 8: "*So, we saw a lot of frozen shoulders coming in, but a lot of them had fixed ideas of what treatment they wanted. They didn't want surgery yet, or they didn't want to take time off work was the other one, but less so. The standout one was they didn't want an operation, or they wanted to try physiotherapy and injection and then they would opt for surgery. They wanted it to be continuum like that, not a one or the other".*

However, surgeons and physiotherapists felt that participants found all trial arms acceptable once they were in the trial.

Surgeon 6: "*Once they had consented to be part of the study, and they had no problems because they were equally welcome on what treatment they would get… all those who once we enrolled them on to the study, were okay with that.*"

Physiotherapists described that participants were surprised with the number of post-procedural physiotherapy sessions they received in the trial.

Physiotherapist 1: "*I think they've been grateful and surprised at the amount of treatment* (Post-procedural physiotherapy) *that they are allowed to have following the procedure."*

For peer review only

DISCUSSION

Principal findings

We interviewed UK FROST participants and healthcare professionals (surgeons and physiotherapists) to understand their experience in the trial. There were several main findings from our study. Participants described that frozen shoulder had a negative impact on all aspects of their life. They reported improved treatment outcomes and satisfaction with the trial treatments. Surgeons and physiotherapists followed a stage-based treatment approach in their routine practice. In addition, they felt that steroids help in reducing pain and allow shoulder exercise, which were beneficial for improving shoulder movements and function. Exercise adherence was considered important for better outcomes. All interviewees had their personal preferences and expectations about UK FROST treatments.

Frozen shoulder impacts all areas of life

Although frozen shoulder has a self-resolving natural history, our findings indicate that it is a painful and debilitating condition causing considerable disability and reduced quality of life in those affected, which are in accord with those reported in previous studies [10, 16-19]. Participants expressed a preference for seeking early diagnosis and referral to a specialist which is again in line with the findings of a previous qualitative study [10]. In the context of variable prognosis (from self-resolving to resistant/chronic cases) of frozen shoulder, a screening tool to identify the sub-group of patients who might benefit from early referral would be helpful. Factors such as severity of symptoms, diabetes, and inability to cope with functional restriction or pain could be incorporated in the model to predict the need for specific treatment.

Pain relief and return of shoulder movements and function are important treatment outcomes

Participants, surgeons, and physiotherapists agreed that pain relief and improvement in function and movement were important treatment outcomes. Their priorities are similar to the results of a previous survey in 225 health care professionals in the UK [20] and three systematic reviews on shoulder outcomes [21-23].

Steroids help in pain relief and to initiate the exercises

Our findings suggest that participants' experiences are in line with the existing evidence on the use of steroid injections to improve pain in frozen shoulder. There is moderate evidence to support the effectiveness of steroids on pain, function and disability compared to placebo [3, 5] and additional benefit with shoulder exercises [24]. Steroids are also reported to be a potentially cost-effective option [21].

Adherence to prescribed exercises is important for better outcomes

Participants and healthcare professionals described continued patient engagement with the prescribed exercise as important for better outcomes [25-27]. During the supervised exercise phase in the trial, UK FROST participants were motivated by treatment benefits [28] and did their home exercises regularly. They were also self-determined [28] to persevere through the pain associated with exercise. However, after the trial, participants did not continue exercising because they thought they achieved enough movement to allow adequate daily function. Therefore, after the trial, they prioritised daily functional activities and ignored occasional pain. These findings are in line with a previous study which conceptualised participants' views on 'ideal' (no symptoms at all) and 'adequate' (return to function with residual deficits) recovery from musculoskeletal complaints [29].

For peer review only

Participants and healthcare professionals had their personal preferences/expectations about UK FROST treatments

It is natural and inevitable that patients have their own treatment preferences. It is well known that personal preferences have a psychological therapeutic impact on treatment outcomes [30, 31]. If participants get their preferred treatment in a trial, they are more likely to stay motivated, comply better and respond well. Participants who do not receive their preferred treatment might lack motivation and become less engaged. Though our findings indicate that participants had mixed preferences before participation, all UK FROST treatments were well-received at the end.

Our interview findings also indicated mixed expectations and preferences among surgeons and physiotherapists, mainly based on their clinical experience [32-34]. Expert surgical and physiotherapy experience require years of skill building, training and repeated practice. It would be highly unlikely if experts did not acquire personal expectations or preferences. Some might argue that this is integral to developing expertise. This has significant implications in trials as it has an important impact on equipoise. Our findings confirm that personal treatment expectations do exist among healthcare professionals, especially when treatment decisions are mainly expertise-based rather than evidence-based in treatment decision-making.

Strengths and limitations

Interviews were conducted by a researcher not involved in UK FROST and by using open-ended questions that allowed interviewees to express their opinions freely. The interview codes and themes were reviewed by an experienced qualitative researcher to ensure rigour of analysis and interpretation of data.

This study has some limitations. Interviews were conducted with patients and healthcare professionals who participated in UK FROST. Therefore, the findings may not be transferable outside this context. Secondly, only two trial participants who did not receive their allocated treatments took part. This could have influenced their predominantly positive experiences with the trial treatments. Thirdly, 93% of the interviews were conducted via telephone. Therefore, we are uncertain if interviewees would have expressed differently in face-to-face interviews. Lastly, those who did not participate in the interviews may have had different experiences.

Implications for clinical practice

Our findings suggest that:

1. Frozen shoulder is a substantially disabling condition. A holistic approach [18] is needed during patient-healthcare professional communication and routine assessments to address the range of problems and to inform patient-centred care.

2. Patients have their own treatment preferences. Healthcare professionals must provide opportunities to address their preferences during the process of shared decision-making.

3. All UK FROST treatments were perceived as acceptable, beneficial, and satisfactory by the trial participants. The benefits and anticipated risks of these treatments must be clearly communicated to the patients during treatment decision-making.

4. Implementation of ESP or PPP programmes within different NHS settings may be challenging. Pilot implementation studies to identify scaling-up strategies would be useful.

For peer review only

CONCLUSION

This qualitative study has provided a fuller understanding of the perspectives of UK FROST trial participants and healthcare professionals. Future trial designs would usefully consider including qualitative research as part of intervention development to ensure feasibility of delivering treatments within the NHS and to guide implementation beyond the trial. Evaluation of hydro-dilation compared to other treatment options is favoured by healthcare professionals. A prognostic tool to assist timely advice and treatment for people at risk of poorer outcomes would be helpful.

For peer review only

FUNDING

This qualitative work was funded as part of the UK FROST Trial by the National Institute for Health Research (NIHR) Health Technology Assessment programme (Project reference 13/26/01).

COMPETING INTERESTS

Cynthia Srikesavan, Francine Toye, Charalambos P. Charalambous declare no competing interests.

Lorna Goodchild, Stephen Brealey, Matthew Northgraves report receiving grant from NIHR Health Technology Assessment programme that funded the trial.

Sarah E Lamb reports grants from the NIHR Health Technology Assessment Programme during the conduct of the study and was a member of several HTA boards from 2010 to 2015 and the NIHR CTU Standing Advisory Committee 2012–2016.

Amar Rangan reports other grants from NIHR HTA, ORUK and H2020 during the conduct of this study; and is a member of the NIHR i4i Challenge Awards Committee (2019-current). South Tees Hospitals NHS Foundation Trust receives educational grant to the department from DePuy Synthes (J&J limited). Institution also receives payment from DePuy Synthes (J&J limited) for Professor Rangan as the co-ordinating Investigator for the GLOBAL ICON Stemless Shoulder System Post Market Clinical Follow Up Study: CT 1401. These are outside and unrelated to the submitted work.

ACKNOWLEDGEMENTS

The authors thank the patient contributors who took part in developing the interview guide and patients and healthcare professionals participated in the interviews. This work was supported by the National Institute for Health Research (NIHR) Oxford Biomedical Research Centre (BRC). The UK FROST team acknowledges the support of the NIHR Clinical Research Network.

CONTRIBUTORS

Cynthia Srikesavan and Francine Toye led on the study and drafted the manuscript. Matthew Northgraves and Stephen Braeley contributed to recruitment. Lorna Goodchild and Sarah E Lamb provided expertise as physiotherapists. Amar Rangan and Charalambos P. Charalambous provided expertise as orthopaedic surgeons. All authors read, provided feedback and approved the final version.

DATA SHARING STATEMENT

All data relevant to the study are included in the article or uploaded as supplementary information. Data are available upon reasonable request.

OPEN ACCESS

This is an open access article distributed in accordance with the Creative Commons Attribution Non Commercial (CC BY-NC 4.0) license, which permits others to distribute, remix, adapt, build upon this work non-commercially, and license their derivative works on different terms, provided the original work is properly cited, appropriate credit is given, any changes made indicated, and the use is non-commercial. See: http:// creative commons.org/ licenses/ by- nc/ 4. 0/.

For peer review only

REFERENCES

1. Rangan A, Gibson J, Brownson P, et al. Frozen shoulder. *Shoulder & elbow*. 2015 Oct; 7(4):299-307.
2. Mezian K, Chang KV. Frozen Shoulder. In: *StatPearls*. Treasure Island (FL): StatPearls Publishing; 2020. Available from: https://www.ncbi.nlm.nih.gov/books/NBK482162/.
3. Rangan A, Hanchard N, McDaid C. What is the most effective treatment for frozen shoulder? *BMJ*. 2016 Aug 23; 354:i4162.
4. Nagy MT, MacFarlane RJ, Yousaf Khan MW. Suppl 3: The Frozen Shoulder: Myths and Realities. *The open orthopaedics journal*. 2013; 7:352.
5. Sun Y, Chen J, Li H, Jiang J, et al. Steroid injection and nonsteroidal anti-inflammatory agents for shoulder pain: a PRISMA systematic review and meta-analysis of randomized controlled trials. *Medicine*. 2015 Dec; 94(50).
6. Le HV, Lee SJ, Nazarian A, Rodriguez EK. Adhesive capsulitis of the shoulder: review of pathophysiology and current clinical interventions. *Shoulder & elbow*. 2017 Apr; 9(2):75-84.
7. Manske RC, Prohaska D. Clinical commentary and literature review: diagnosis, conservative and surgical management of adhesive capsulitis. *Shoulder & Elbow*. 2010 Oct; 2(4):238-54.
8. Sharma S, Jacobs L. Management of frozen shoulder–conservative vs surgical? *The Annals of The Royal College of Surgeons of England*. 2011 Jul; 93(5):343-343.
9. Carter B. Clients' experiences of frozen shoulder and its treatment with Bowen technique. *Complement Ther Nurs Midwifery* 2002; 8:204–10.
10. Jones S, Hanchard N, Hamilton S, Rangan A. A qualitative study of patients' perceptions and priorities when living with primary frozen shoulder. *BMJ open*. 2013 Sep 1; 3(9):e003452.
11. Brealey S, Armstrong AL, Brooksbank A, et al. United Kingdom Frozen Shoulder Trial (UK FROST), multi-centre, randomised, 12 month, parallel group, superiority study to compare the clinical and cost-effectiveness of Early Structured Physiotherapy versus manipulation under anaesthesia versus arthroscopic capsular release for trial participants referred to secondary care with a primary frozen shoulder: study protocol for a randomised controlled trial. *Trials*. 2017 Dec; 18(1):614.
12. Charmaz, K. (2006). Constructing Grounded Theory – A Practical Guide through Qualitative Analysis. SAGE.
13. Rindflesch AB. A grounded-theory investigation of patient education in physical therapy practice. *Physiotherapy theory and practice*. 2009 Jun; 25(3):193-202.
14. NVivo qualitative data analysis Software; QSR International Pty Ltd. Version 11, 2012.
15. O'Brien BC, Harris IB, Beckman TJ, Reed DA, Cook DA. Standards for reporting qualitative research: a synthesis of recommendations. *Academic Medicine*. 2014 Sep 1; 89(9):1245-51.
16. Roe Y, Bautz-Holter E, Juel NG, et al. Identification of relevant International Classification of Functioning, Disability and Health categories in patients with shoulder pain: a cross-sectional study. *J Rehabil Med* 2013;45:662-9. http://dx.doi.org/10.2340/16501977-1159.
17. Bagheri F, Ebrahimzadeh MH, Moradi A, et al. Factors Associated with Pain, Disability and Quality of Life in Patients Suffering from Frozen Shoulder. *Arch Bone Jt Surg* 2016;**4**:243-7.
18. Toprak M, Erden M. Sleep quality, pain, anxiety, depression and quality of life in patients with frozen shoulder1. *J Back Musculoskelet Rehabil* 2019;32:287-91. http://dx.doi.org/10.3233/bmr-171010.
19. Page MJ, O'Connor DA, Malek M, et al. Patients' experience of shoulder disorders: a systematic review of qualitative studies for the OMERACT Shoulder Core Domain Set. *Rheumatology* (Oxford) 2019; 10.1093/rheumatology/kez046. http://dx.doi.org/10.1093/rheumatology/kez046.

20. Rodgers S, Brealey S, Jefferson L, et al. Exploring the outcomes in studies of primary frozen shoulder: is there a need for a core outcome set? *Quality of Life Research.* 2014 Nov 1; 23(9):2495-504.
21. Maund E, Craig D, Suekarran S, et al. Management of frozen shoulder: a systematic review and cost-effectiveness analysis. *Health Technology Assessment* (Winchester, England). 2012; 16(11):1.
22. Page MJ, Huang H, Verhagen AP, et al. Outcome reporting in randomized trials for shoulder disorders: Literature review to inform the development of a core outcome set. *Arthritis care & research.* 2018 Feb; 70(2):252-9.
23. Page MJ, McKenzie JE, Green SE, et al. Core domain and outcome measurement sets for shoulder pain trials are needed: systematic review of physical therapy trials. *Journal of clinical epidemiology*. 2015 Nov 1; 68(11):1270-81.
24. Page MJ, Green S, Kramer S, et al. Manual therapy and exercise for adhesive capsulitis (frozen shoulder). *Cochrane Database Syst Rev* 2014; 10.1002/14651858.cd011275:Cd011275.
25. Bailey DL, Holden MA, Foster NE, et al. Defining adherence to therapeutic exercise for musculoskeletal pain: a systematic review. *Br J Sports Med* 2018; 10.1136/bjsports-2017-098742. http://dx.doi.org/10.1136/bjsports-2017-098742.
26. Chester R, Jerosch-Herold C, Lewis J, et al. Psychological factors are associated with the outcome of physiotherapy for people with shoulder pain: a multicentre longitudinal cohort study. *Br J Sports Med* 2018;52:269-75. http://dx.doi.org/10.1136/bjsports-2016-096084.
27. Picorelli AM, Pereira LS, Pereira DS, et al. Adherence to exercise programs for older people is influenced by program characteristics and personal factors: a systematic review. *J Physiother* 2014;60:151-6. http://dx.doi.org/10.1016/j.jphys.2014.06.012.
28. Teixeira PJ, Carraca EV, Markland D, et al. Exercise, physical activity, and self-determination theory: a systematic review. *Int J Behav Nutr Phys Act* 2012;9:78. http://dx.doi.org/10.1186/1479-5868-9-78.
29. Carroll LJ, Lis A, Weiser S, Torti J. How Well Do You Expect to Recover, and What Does Recovery Mean, Anyway? Qualitative Study of Expectations After a Musculoskeletal Injury. *Phys Ther* 2016;96:797-807. http://dx.doi.org/10.2522/ptj.20150229.
30. Preference Collaborative Review Group. Patients' preferences within randomised trials: systematic review and patient level meta-analysis. *BMJ* 2008;337: a1864. http://dx.doi.org/10.1136/bmj.a1864.
31. Walter SD, Turner RM, Macaskill P, et al. Estimation of treatment preference effects in clinical trials when some participants are indifferent to treatment choice. *BMC Med Res Methodol* 2017;17:29. http://dx.doi.org/10.1186/s12874-017-0304-x.
32. Cook C, Sheets C. Clinical equipoise and personal equipoise: two necessary ingredients for reducing bias in manual therapy trials. *J Man Manip Ther* 2011;19:55-7. http://dx.doi.org/10.1179/106698111x12899036752014.
33. Ghogawala Z, Barker FG, Carter BS. Clinical equipoise and the surgical randomized controlled trial. *Neurosurgery*. 2008 Jun 1; 62(6):N9-10.
34. Kwaees TA, Charalambous CP. Surgical and non-surgical treatment of frozen shoulder. Survey on surgeons treatment preferences. *Muscles Ligaments Tendons J* 2014;4:420-4.

FIGURE LEGENDS

Figure 1: Interviews with trial participants. ESP: Early Structured Physiotherapy; MUA: Manipulation Under Anaesthesia; ACR: Arthroscopic Capsular Release.

Figure 2: Interviews with surgeons and physiotherapists.

For peer review only

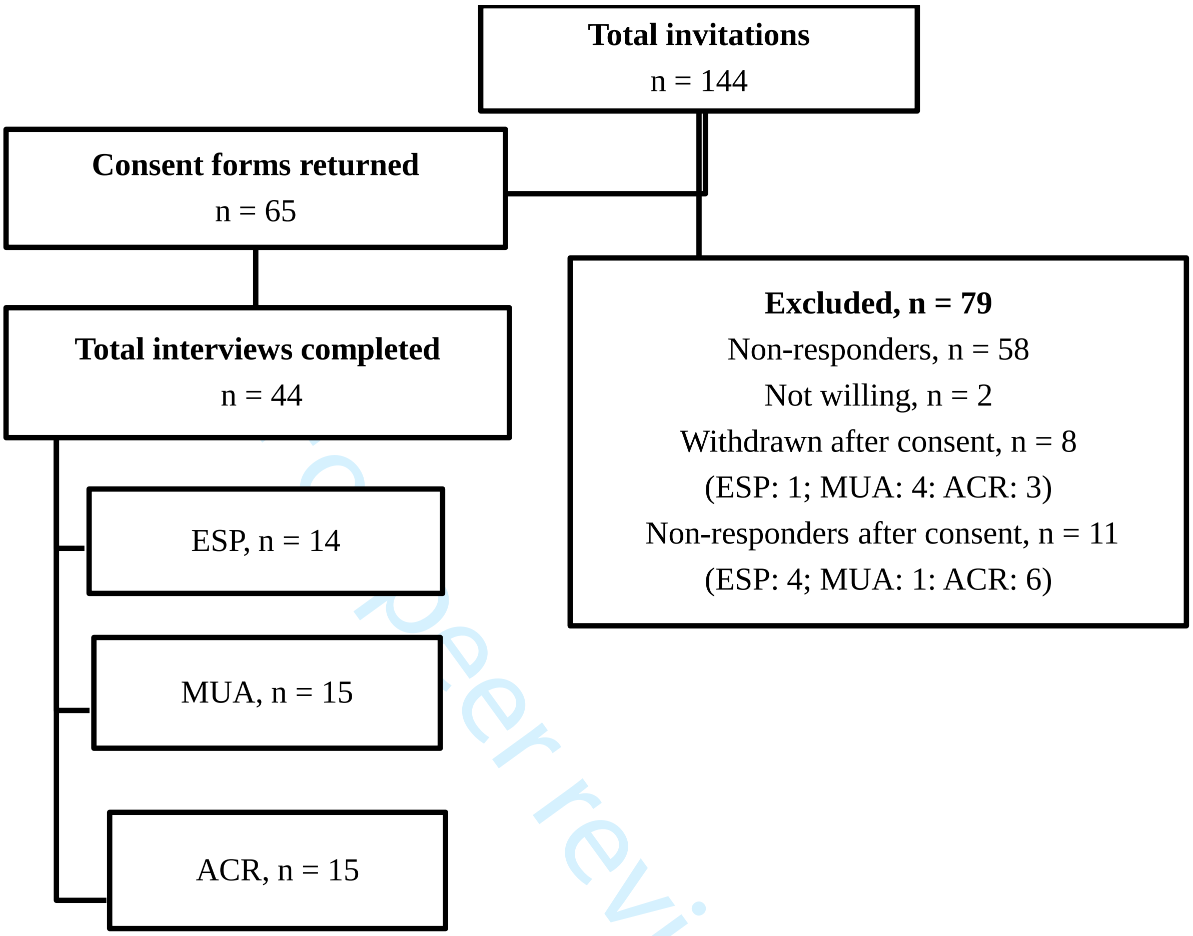

Figure 1: Interviews with trial participants. ESP: Early Structured Physiotherapy; MUA: Manipulation Under Anaesthesia; ACR: Arthroscopic Capsular Release.

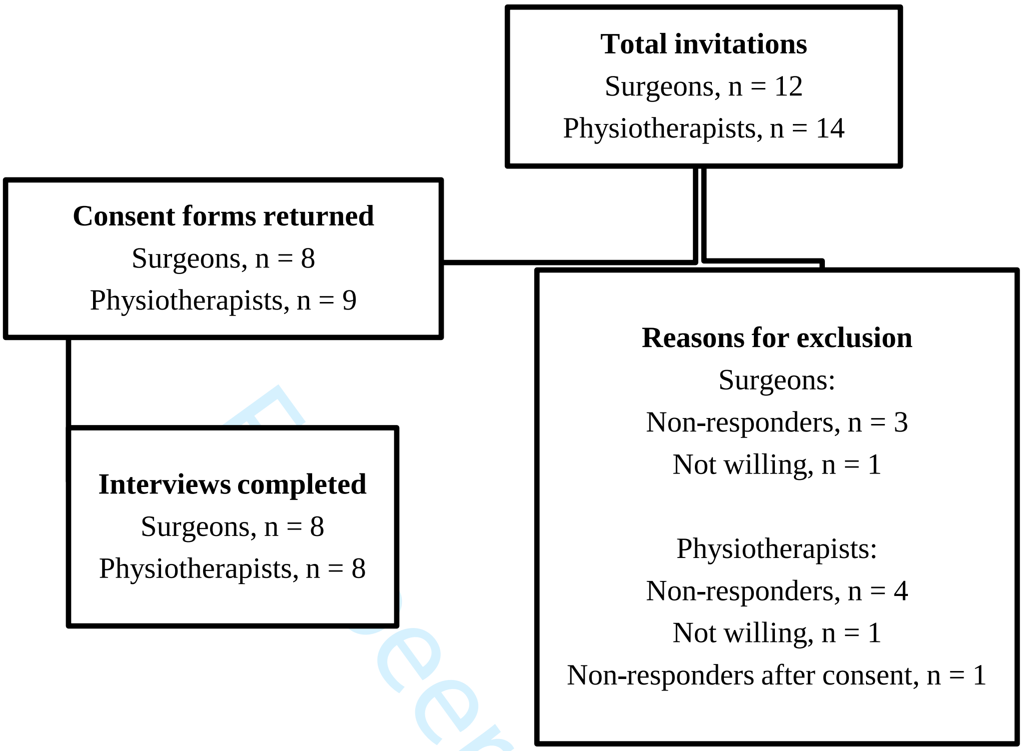

Figure 2: Interviews with surgeons and physiotherapists.

For peer review only

Supplementary file: Participant-reported problems due to frozen shoulder

a. Participant-reported impairments

Trial participants' narratives	Impairments
"It was like a stabbing pain; it was very severe" (ACR)	Pain
"I couldn't move my shoulder at all. So it was stuck to my side" (ESP)	Reduced joint mobility
"Definitely lost my strength in my arm" (MUA)	Reduced muscle strength
"I didn't sleep at all" (ESP)	Sleep disturbances
"I was generally getting very depressed. I would have happily, towards the end, I would happily have them amputate the arm" (MUA)	Psychological problems

For peer review only

b. Participant-reported problems in activities of daily life and social participation

Trial participants' narratives	Activities of daily life & social participation
"Carrying was probably a bit of a problem because I couldn't move that arm so well" (ESP)	Carrying objects
"Reaching things from the tall shelves in the kitchen, reaching stuff out of the top of the wardrobe…And its things you take for granted really" (MUA)	Reaching objects
"I couldn't drive my car even. I couldn't change gear"(ESP)	Driving
"I couldn't lift my hand up, my arm up, you know, to wash my hair or anything in the bath. And eventually I couldn't get into the bath properly" (ESP)	Washing oneself
"It was the pain and the stiffness, particularly the stiffness, and the inability to address and attend to myself at the toilet. It was becoming a personal hygiene issue for me" (ACR)	Toileting
"I couldn't get myself dressed. I couldn't get my tops on above my head. I had to wear slack things, so I could get my clothes on" (ACR)	Dressing
"I couldn't open anything, I couldn't use a tin opener, so I couldn't cook neither, I couldn't do anything" (ACR)	Preparing meals
"Doing housework was nigh impossible"(MUA)	Housework
"I'm a coachbuilder by trade…I couldn't work overhead, I couldn't lift my arms up and I couldn't stretch my arms out, I just couldn't do it, so I wasn't, I didn't go to work, I was off for about seven months". (MUA)	Employment
"Well I decorate cakes. I do novelty cakes, all kinds of decorating. That literally stopped" (ACR)<br>*"I like gardening and I was doing the decorating on my house, all sorts of jobs like that, sport, tennis, anything like that it sort of ruined everything really"* (MUA)	Recreation and leisure
"I couldn't sleep on my left side anymore and if I turned over in the night and tried to sleep on that side it instantly woke me up." (MUA)	Changing body position

Reporting checklist for qualitative study.

Based on the SRQR guidelines.

Instructions to authors

Complete this checklist by entering the page numbers from your manuscript where readers will find each of the items listed below.

Your article may not currently address all the items on the checklist. Please modify your text to include the missing information. If you are certain that an item does not apply, please write "n/a" and provide a short explanation.

Upload your completed checklist as an extra file when you submit to a journal.

In your methods section, say that you used the SRQRreporting guidelines, and cite them as:

O'Brien BC, Harris IB, Beckman TJ, Reed DA, Cook DA. Standards for reporting qualitative research: a synthesis of recommendations. Acad Med. 2014;89(9):1245-1251.

		Reporting Item	Page Number
**Title**			
	#1	Concise description of the nature and topic of the study identifying the study as qualitative or indicating the approach (e.g. ethnography, grounded theory) or data collection methods (e.g. interview, focus group) is recommended	1
**Abstract**			
	#2	Summary of the key elements of the study using the abstract format of the intended publication; typically includes background, purpose, methods, results and conclusions	2
**Introduction**			
Problem formulation	#3	Description and significance of the problem / phenomenon studied: review of relevant theory and empirical work; problem statement	4
Purpose or research	#4	Purpose of the study and specific objectives or	4

For peer review only

question		questions	
**Methods**			
Qualitative approach and research paradigm	#5	Qualitative approach (e.g. ethnography, grounded theory, case study, phenomenolgy, narrative research) and guiding theory if appropriate; identifying the research paradigm (e.g. postpositivist, constructivist / interpretivist) is also recommended; rationale. The rationale should briefly discuss the justification for choosing that theory, approach, method or technique rather than other options available; the assumptions and limitations implicit in those choices and how those choices influence study conclusions and transferability. As appropriate the rationale for several items might be discussed together.	5
Researcher characteristics and reflexivity	#6	Researchers' characteristics that may influence the research, including personal attributes, qualifications / experience, relationship with participants, assumptions and / or presuppositions; potential or actual interaction between researchers' characteristics and the research questions, approach, methods, results and / or transferability	4
Context	#7	Setting / site and salient contextual factors; rationale	4
Sampling strategy	#8	How and why research participants, documents, or events were selected; criteria for deciding when no further sampling was necessary (e.g. sampling saturation); rationale	4, 5
Ethical issues pertaining to human subjects	#9	Documentation of approval by an appropriate ethics review board and participant consent, or explanation for lack thereof; other confidentiality and data security issues	4
Data collection methods	#10	Types of data collected; details of data collection procedures including (as appropriate) start and stop dates of data collection and analysis, iterative process, triangulation of sources / methods, and modification of procedures in response to evolving	4

For peer review only

		study findings; rationale	
Data collection instruments and technologies	#11	Description of instruments (e.g. interview guides, questionnaires) and devices (e.g. audio recorders) used for data collection; if / how the instruments(s) changed over the course of the study	4
Units of study	#12	Number and relevant characteristics of participants, documents, or events included in the study; level of participation (could be reported in results)	Figure 1 & 2
Data processing	#13	Methods for processing data prior to and during analysis, including transcription, data entry, data management and security, verification of data integrity, data coding, and anonymisation / deidentification of excerpts	4
Data analysis	#14	Process by which inferences, themes, etc. were identified and developed, including the researchers involved in data analysis; usually references a specific paradigm or approach; rationale	5
Techniques to enhance trustworthiness	#15	Techniques to enhance trustworthiness and credibility of data analysis (e.g. member checking, audit trail, triangulation); rationale	5
**Results/findings**			
Syntheses and interpretation	#16	Main findings (e.g. interpretations, inferences, and themes); might include development of a theory or model, or integration with prior research or theory	5-12
Links to empirical data	#17	Evidence (e.g. quotes, field notes, text excerpts, photographs) to substantiate analytic findings	5-12, Supplementary file
**Discussion**			
Intergration with prior work, implications, transferability and contribution(s) to the field	#18	Short summary of main findings; explanation of how findings and conclusions connect to, support, elaborate on, or challenge conclusions of earlier scholarship; discussion of scope of application / generalizability; identification of unique contributions(s) to scholarship in a discipline or field	13

For peer review only

Limitations	#19	Trustworthiness and limitations of findings	14
**Other**			
Conflicts of interest	#20	Potential sources of influence of perceived influence on study conduct and conclusions; how these were managed	16
Funding	#21	Sources of funding and other support; role of funders in data collection, interpretation and reporting	16

None The SRQR checklist is distributed with permission of Wolters Kluwer © 2014 by the Association of American Medical Colleges. This checklist can be completed online using https://www.goodreports.org/, a tool made by the EQUATOR Network in collaboration with Penelope.ai

For peer review only

BMJ Open

The experiences and perceptions of trial participants and healthcare professionals in the United Kingdom Frozen Shoulder Trial (UK FROST): A nested qualitative study.

Journal:	*BMJ Open*
Manuscript ID	bmjopen-2020-040829.R1
Article Type:	Original research
Date Submitted by the Author:	07-Feb-2021
Complete List of Authors:	Srikesavan, Cynthia; University of Oxford, Nuffield Department of Orthopaedics, Rheumatology, and Musculoskeletal Sciences<br>Toye, Francine; Oxford University Hospitals NHS Foundation Trust Nuffield Orthopaedic Centre, Physiotherapy Research Unit<br>Brealey, Stephen; University of York, York Trials Unit, ARRC Building, Department of Health Sciences.<br>Goodchild, Lorna; The Physiotherapy Practice<br>Northgraves, Matthew; Hull York Medical School<br>Charalambous, C; Blackpool Teaching Hospitals NHS Foundation Trust; University of Central Lancashire, School of Medicine<br>Rangan, Amar; University of York, York Trials Unit, ARRC Building, Department of Health Sciences<br>Lamb, Sarah; University of Exeter, College of Medicine and Health; University of Oxford, Nuffield Department of Orthopaedics, Rheumatology, and Musculoskeletal Sciences
**Primary Subject Heading**:	Qualitative research
Secondary Subject Heading:	Qualitative research
Keywords:	Elbow & shoulder < ORTHOPAEDIC & TRAUMA SURGERY, Musculoskeletal disorders < ORTHOPAEDIC & TRAUMA SURGERY, Shoulder < ORTHOPAEDIC & TRAUMA SURGERY

SCHOLARONE™ Manuscripts

I, the Submitting Author has the right to grant and does grant on behalf of all authors of the Work (as defined in the below author licence), an exclusive licence and/or a non-exclusive licence for contributions from authors who are: i) UK Crown employees; ii) where BMJ has agreed a CC-BY licence shall apply, and/or iii) in accordance with the terms applicable for US Federal Government officers or employees acting as part of their official duties; on a worldwide, perpetual, irrevocable, royalty-free basis to BMJ Publishing Group Ltd ("BMJ") its licensees and where the relevant Journal is co-owned by BMJ to the co-owners of the Journal, to publish the Work in this journal and any other BMJ products and to exploit all rights, as set out in our licence.

The Submitting Author accepts and understands that any supply made under these terms is made by BMJ to the Submitting Author unless you are acting as an employee on behalf of your employer or a postgraduate student of an affiliated institution which is paying any applicable article publishing charge ("APC") for Open Access articles. Where the Submitting Author wishes to make the Work available on an Open Access basis (and intends to pay the relevant APC), the terms of reuse of such Open Access shall be governed by a Creative Commons licence – details of these licences and which Creative Commons licence will apply to this Work are set out in our licence referred to above.

Other than as permitted in any relevant BMJ Author's Self Archiving Policies, I confirm this Work has not been accepted for publication elsewhere, is not being considered for publication elsewhere and does not duplicate material already published. I confirm all authors consent to publication of this Work and authorise the granting of this licence.

For peer review only

Title page

Title of the article

The experiences and perceptions of trial participants and healthcare professionals in the United Kingdom Frozen Shoulder Trial (UK FROST): A nested qualitative study.

Full name, postal address and e-mail of the corresponding author

Dr Francine Toye (Ph.D, M.A Archaeology & Anthropology Cantab)
Qualitative Research Lead, Physiotherapy Research Unit,
Nuffield Orthopaedic Centre,
Oxford University Hospitals NHS Foundation Trust,
Windmill Road, Oxford OX3 7HE.
United Kingdom.

Email: francine.toye@ouh.nhs.uk.

Full name, department, institution, city and country of all co-authors
[1]Cynthia Srikesavan, [2]Francine Toye, [3]Stephen Brealey, [4]Lorna Goodchild, [5]Matthew Northgraves, [6]Charalambos P. Charalambous, [7]Amar Rangan, [8]Sarah E Lamb.

Affiliations
[1]Nuffield Department of Orthopaedics, Rheumatology, and Musculoskeletal Sciences, University of Oxford, Windmill road, Oxford, United Kingdom.
[2]Nuffield Orthopaedic Centre, Oxford University Hospitals NHS Foundation Trust, Windmill Road, Oxford, United Kingdom.
[3, 7]York Trials Unit, ARRC building, Department of Health Sciences, University of York, ARRC Building, York, United Kingdom.
[4]The Physiotherapy Practice, South Shields, United Kingdom.
[5]Hull York Medical School, Hull, United Kingdom.
[6]Blackpool Teaching Hospitals NHS Trust, Blackpool, and School of Medicine, University of Central Lancashire, Preston, United Kingdom.
[8]College of Medicine and Health, University of Exeter & Nuffield Department of Orthopaedics, Rheumatology, and Musculoskeletal Sciences, University of Oxford, Windmill road, Oxford, United Kingdom.

Word count, excluding title page, abstract, references, figures and tables: 4697

Keywords: Adhesive capsulitis; Arthroscopic capsular release; Frozen shoulder; Interviews; Manipulation under anaesthesia; Physiotherapy.

Abstract

Abstract word count: 298

Objectives

To explore the experiences and perceptions of trial participants and healthcare professionals in the United Kingdom Frozen Shoulder Trial (UK FROST), a multi-centre randomised controlled trial that compared manipulation under anaesthesia (MUA), arthroscopic capsular release (ACR) with a 12-week early structured physiotherapy programme (ESP) in people with unilateral frozen shoulder referred to secondary care.

Design

Nested qualitative study with semi-structured interviews. We used constant comparison method to develop our themes.

Setting

Face-to-face or telephone interviews.

Participants

44 trial participants (ESP: 14; MUA: 15; ACR: 15), and 8 surgeons and 8 physiotherapists who delivered the treatments in the trial.

Results

Trial participants found UK FROST treatments acceptable and satisfactory in terms of content, delivery and treatment benefits. Participants in all arms experienced improvements in pain, shoulder movements, and function. Participants said they would choose the same treatment that they received in the trial.

Surgeons and physiotherapists felt that the content and delivery of UK FROST treatments was not significantly different to their routine practice except for the additional number of physiotherapy sessions offered in the trial. They had mixed feelings about the effectiveness of UK FROST treatments. Both stressed the value of including hydro-dilatation as a comparator of other treatment options. Physiotherapists raised concerns about the capacity to deliver the number of UK FROST physiotherapy sessions in routine clinical settings.

Shared perceptions of trial participants, surgeons and physiotherapists were: 1) Pain relief and return of shoulder movements and function are important outcomes; and 2) Adherence to exercises leads to better outcomes.

Conclusion

In general, our findings indicated that trial participants, and surgeons and physiotherapists who delivered the treatments had positive experiences and perceptions in the UK FROST. Early qualitative investigations to explore the feasibility of delivering treatments in real-world settings are suggested in future trials in frozen shoulder.

For peer review only

Keywords: Adhesive capsulitis; Arthroscopic capsular release; Frozen shoulder; Interviews; Manipulation under anaesthesia; Physiotherapy.

ARTICLE SUMMARY

Strengths and limitations of this study

- Interviews were conducted and analysed by a researcher who was not involved in the UK FROST.
- Interview codes and themes were reviewed by an experienced researcher to ensure rigour of analysis and interpretation of data.
- Interviews were conducted with participants and healthcare professionals who participated in the UK FROST. Therefore, the findings may not be transferable outside this context.
- 93% of the interviews were telephone-based. It is uncertain if interviewees would have expressed differently in face-to-face interviews.
- Those who did not participate in the interviews may have had different experiences.

For peer review only

Main text word count: 4227 (excluding Tables and Footnotes)

INTRODUCTION

Frozen shoulder or adhesive capsulitis is a chronic shoulder condition commonly affecting people aged between 50 and 60 years [1]. Women are affected four times than men [2], while the prevalence rate is around 20% in people with diabetes [2]. Frozen shoulder is characterised by three clinical stages that often overlap [3, 4]: 1) a painful stage (3 to 8 months), 2) a stiffness (frozen) stage (4 to 12 months) with progressive loss of active and passive shoulder movements and 3) a thawing stage (12 to 42 months) with gradual recovery of movements in most people [2].

Frozen shoulder is managed by non-surgical or surgical treatments [3, 5-9]. However, there is no high-quality evidence to recommend one treatment over another [3]. The United Kingdom Frozen Shoulder Trial (UK FROST) is a multi-centre, pragmatic randomised controlled trial in adults with frozen shoulder [10]. It evaluated the clinical and cost-effectiveness of two common surgical treatments: manipulation under anaesthesia (MUA) and arthroscopic capsular release (ACR) both followed by up to 12 weeks of post-procedural physiotherapy (PPP), with a 12-week early structured physiotherapy (ESP) programme that included an intra-articular steroid injection. Detailed descriptions of the trial treatments, including the specifically developed ESP and PPP pathways for the trial are available [10].

The qualitative literature on frozen shoulder is limited [11, 12]. There are no trials in frozen shoulder with an embedded qualitative component that explored the perspectives of trial participants (patients with frozen shoulder) and healthcare professionals who delivered the trial treatments. Therefore, we proposed a nested qualitative study within the UK FROST to explore the experiences and perceptions of trial participants who received UK FROST treatments, and healthcare professionals (surgeons and physiotherapists) who delivered those treatments.

METHODS

Ethics approval was obtained from the NRES Committee North East -Newcastle and North Tyneside 2 ethics committee (14/NE/1176) and Health Research Authority (HRA).

A qualitative and constructivist approach [13] was adopted to understand the experiences and perceptions of trial participants and healthcare professionals in the UK FROST.
The primary author (CS) is a physiotherapy researcher trained in qualitative methods and not involved in the delivery of UK FROST treatments and outcome measurement. CS conducted all the interviews and led the analysis. FT is an experienced qualitative researcher, anthropologist and physiotherapist, also not involved in delivery of care or outcome measurement. FT played a collaborative role in analysis.

For peer review only

Participants who took part in the main trial had primary unilateral frozen shoulder, identified through a restriction of passive external rotation on clinical examination. They were randomly assigned to MUA, ACR or ESP treatment arms in the ratio of 2:2:1. A purposive sample of trial participants was recruited from those who had agreed to be contacted by the trial team for interviews at approximately 12 months post-randomisation, which coincided with the primary endpoint of the trial and completion of UK FROST treatments [10]. We proposed to recruit up to 45 trial participants. As gender and diabetes are likely to have an impact on the treatment outcomes [2, 10], we also planned to include an equal number of men and women, and those with and without diabetes.

We also planned to recruit a purposive sample of up to 15 surgeons and physiotherapists who delivered the treatments in the trial and agreed to take part in the interviews.

The study information sheet and consent form were posted to potential trial participants and emailed to surgeons and physiotherapists. Two postal or email reminders were sent to those who didn't respond within four weeks of invitation. Once signed consent was received via post or email from volunteer participants, surgeons and physiotherapists, CS contacted potential participants via telephone to coordinate an interview appointment with them.

Separate semi-structured interview guides with open-ended questions were used for trial participants and health professionals. Given the geographical spread of those participated, interviews were conducted face-to-face or via telephone. Interviews were audio-recorded using a digital recorder, anonymised and transcribed verbatim by a professional transcription agency.

The interviews were analysed using constant comparative method [13, 14] to develop categories and themes with a shared essence. The QSR International's NVivo 11 software [15] was used to organise and assist analysis of the data. The analysis was stopped at the point where additional data no longer contributed to the understanding of the themes. To enhance credibility of the findings, codes and themes were reviewed by an experienced qualitative researcher (FT). The standards for reporting qualitative research (SRQR) guidelines were used to report the study [16].

Patient and public involvement

The patient interview guide was developed following discussions with patients with frozen shoulder, research team, a physiotherapist and surgeon with expertise in this area, and reviewing the literature.

RESULTS

Sixty interviews (Participants: 44; Surgeons: 8 and Physiotherapists: 8) were completed between August 2016 and January 2018. Most interviews (56/60, 93%) were conducted via

For peer review only

telephone. Four interviews were held at the clinic offices of surgeons and physiotherapists. Though constant comparison was used to analyse data, a formal theory was not developed.

A. Interviews with trial participants

A flow diagram showing the number of patients invited, excluded and participated in the interviews is presented in Figure 1. Participant characteristics are presented in Table 1. Apart from two participants, all other participants received their allocated treatment.

Five themes were identified. The themes were not different between men and women; and between those with and without diabetes.

Table 1: Characteristics of the participants interviewed

UK FROST arms	Male	Female	Age<br>Median (IQR) in years	Diabetic	Non-diabetic
ESP	5	9	58 (51 to 63.5)	3	11
MUA	8	7	55 (53 to 57.5)	5	10
ACR	7	8	59 (53 to 69)	5	10

UK FROST: United Kingdom Frozen Shoulder Trial; IQR: Inter-quartile range; ESP: Early Structured Physiotherapy; MUA: Manipulation Under Anaesthesia; ACR: Arthroscopic Capsular Release.

1) Experiences and perceptions of participating in the UK FROST

In general, trial participants had positive experiences in participating in UK FROST. They described altruistic and personal reasons for taking part in the trial. They were concerned about their upper limb disability from frozen shoulder and were keen on getting it sorted in UK FROST. There was also an overall sense that the trial questionnaires were relevant, simple and straightforward to complete.

> *"Because I'm all in favour, if you can do something to help other people not go through the misery that you've been through, and gone through, then I would do it".* (ESP)

For peer review only

Trial participants felt that their treatment packages were well coordinated and did not require any modification. The surgical procedures were clearly explained and physiotherapists who delivered ESP and PPP were supportive and helpful. Participants also liked to see the same physiotherapist for developing good connections and rapport throughout the study period. However, some ESP and ACR arm participants felt that the exercises were time consuming and found it difficult to fit into their daily life. Some said that they would have liked more information about continuing exercises after the trial and exercising when in pain.

> *"Operation, I believe obviously went fantastic, went really well. It was just a case of waking up, recovery*."(ACR)

> *"Well personally I found it quite difficult to make sure I had them that many times in the day. I'm not too sure how somebody working or having a family could actually manage to fit it in because as I say, by the time going towards the middle to the end of the programme…"* (ESP)

2) Acceptability - Treatment satisfaction and improved outcomes

All UK FROST treatments were found to be acceptable, satisfactory and beneficial. Except for two participants in the ESP arm, others were satisfied with the UK FROST treatments they received. Of these two exceptions, one did not improve after physiotherapy in the trial and the other was not satisfied when the exercise sessions were supervised by an unfamiliar physiotherapist.

"*I'm absolutely delighted with the treatment that I was given. I feel as though it did everything that I wanted it to do and expected it to do"*. (ESP)

"*Very satisfied. I have no complaints at all"* (ACR).

Participants considered pain relief and return of shoulder movements and function as important treatment outcomes. Participants in all arms experienced improvements in pain, movements and function. In spite of achieving pain relief and improved function, some participants said they experienced mild and occasional pain and restrictions during certain end-range activities. However, this did not impact their daily functioning.

Pain:
Trial participants experienced a substantial reduction in pain in all treatment arms. The ESP arm participants said that the steroid injections reduced pain and allowed them to start physiotherapy.

> *"When I went to the surgeon I was injected into my shoulder and the pain down my arm that more or less went straightaway."* (ESP)

For peer review only

"So at the beginning I said the pain was ten and now after all my physios, I'd say it was, I'd say it was about two now." (MUA)

"I mean the pain in the beginning was just horrendous, it was really, really sore, really painful but after I'd had the physiotherapy, it was… I've got no pain at all now." (ACR)

Movements:

Trial participants described how the physiotherapy sessions (ESP and post-procedural) had helped to improve their shoulder movements.

"I could tell initially straightaway that my movement was starting to come, within a few days I could tell a difference of doing the exercises and as the weeks went on, it was just got better and better and by the time the twelve weeks was up, I virtually had full movements with no pain or anything, it was brilliant!" (ESP)

"After a few days I was doing my exercises and I was quite surprised already how much movement I had back and then it was regular physio appointments up at the hospital just to keep moving things around and that went really well…the physiotherapy was actually really, really beneficial." (MUA)

In the ACR arm, improvement in movements was thought to be quicker than participants had expected. Some experienced improvements as early as one to two weeks of physiotherapy after surgery.

"*I felt that the physiotherapy I received was marvellous and improved the range of movements or showed me how to keep that range of movements much quicker than they did on the right-hand side, so I felt that everything went along fine, and I've got no complaints at all, none*." (ACR)

"*It is almost like you have had a quick fix to fix your shoulder then you move on and I think personally for me because the surgery went very well and almost after a couple of weeks I was back to normal*." (ACR)

Function:

Trial participants in all treatment arms described how their ability to do routine activities improved.

"I can lift my arm above my head now, you know? I can carry stuff, and I can lift it above my waist, and I can actually go swimming, you know? I can swim now." (ESP)

"My little everyday things have come back; I have come back, yes". (MUA)

For peer review only

"I can do everything – there's nothing that I can't do; I can wash my back, I can put my bra on, fasten it at the back, I can fasten my skirt at the side and the back now, there's nothing I can't do before I had the frozen shoulder everything I could do then I can now do again". (ACR)

3) Adherence to exercises

Trial participants described that ESP and PPP were difficult to begin with, but became easier on subsequent sessions.

"It (Physiotherapy) *was agony but from every session there was slight improvement, so for every kind, every session I had, I had more movement and less pain, so it got to a point where some days I would go down and I had no pain at all."* (ESP)

However, participants were aware of the benefits of exercises and persevered with their home exercises during the study period.

"It was uncomfortable but I tried really hard with them to be honest because it was so awful when I couldn't use my arm that I knew that the only way to help it was to do them as much as I could." (MUA)

Several participants said they did not continue their home exercises after the trial because they felt they had regained adequate shoulder function. Instead, they maintained their shoulder mobility by being functionally active. A few participants occasionally did some shoulder stretches.

"*I'm working with my shoulder all the time so I'm not doing the exercises that the hospital gave me, because I'm working my, I'm swimming, I'm doing…I go on long walks, I take the dog out and what have you, so I'm using my arm.*" (ESP)

4) Personal treatment preferences

Trial participants had mixed treatment preferences before the trial. Some considered physiotherapy to be ineffective, while a few wanted to avoid the risks of surgery. Some preferred the less invasive MUA while some thought ACR as the final solution. Some didn't have any preference at all.

"I'm not really too sure why I wouldn't choose physiotherapy. I just think surgery seems a more final option. Physiotherapy, it might work, it may help, it may not. But to me, if I was given surgery, the surgery would work. I had more faith in the surgery working than the physiotherapy itself." (ACR)

"Well I didn't want to go to surgery or anything like that, so I just had the needle in my shoulder. I don't think I'd have wanted to go surgery at my age" (ESP)

For peer review only - http://bmjopen.bmj.com/site/about/guidelines.xhtml

Despite preferences at the outset, at the end of the trial, there was a sense that participants would choose the same treatment they received in the UK FROST. In retrospect, some participants in ESP and MUA said that they would choose surgery as a permanent and time-saving solution for their shoulder problem.

> *"Oh no I would have the same treatment. As I say I was only there for ten weeks and I mean, on my eleventh week I was still hell of a lot better."* (ESP)

B. Interviews with surgeons and physiotherapists

A flow diagram showing the number of health professionals invited, excluded and participated in the interviews is presented in Figure 2. The characteristics of surgeons and physiotherapists are presented in Table 2.

Four themes were identified.

Table 2: Characteristics of the surgeons and physiotherapists interviewed

	**Gender**		**Years of experience in treating shoulder conditions**
	Male	Female	Median (IQR) years
Surgeons	7	1	11 (7 to16.25)
Physiotherapists	--	8	13.5 (12 to 15.75)

IQR: Inter-quartile range

1) Experiences and perceptions of delivering UK FROST treatments

In general, surgeons and physiotherapists had positive experiences in delivering the trial treatments. Surgeons described how the surgical treatments were no different from their usual practice. They described how trial participants who received MUA or ACR were usually seen by specialised physiotherapists for advice and PPP at the follow-up stage. Some said there were no reports of complications or side-effects due to surgery, whilst some said that a few patients did not respond well to ESP.

> Surgeon 01: *"Technically there was no difference, I wouldn't do anything different".*

For peer review only

Surgeon 06: *"In the physiotherapy group, there have been a couple of patients who have not responded very well to surgery, very well to the treatment, to the physiotherapy treatment and we have just carried on and they've taken a bit longer."*

Physiotherapists felt that the UK FROST physiotherapy programmes and the exercise booklet gave more flexibility in choosing the exercises than they would usually have in their routine practice. The only difference was the increased number of sessions offered in the trial. Surgical soreness was a commonly seen post-operative issue in ACR and MUA participants. However, it was quick to resolve with PPP compared to those who received ESP. There were a few suggestions from physiotherapists to improve the UK FROST exercise intervention. For example, one physiotherapist felt the exercises could have been standardised better. Two others suggested either spreading the 12 weekly sessions over 6 months or doing group sessions.

Physiotherapist 1: "I *think the interventions that were on the booklet were what I would use generally. There was always an option there for me to tick off what I would do so I was in agreement with the options that were there and in agreement with the options that they actually, didn't want you to use*".

Physiotherapist 5: "*I think group sessions would be really useful because patients get a lot from each other, and having experience group sessions with other clients with different pathologies, you know, they find that really reassuring…*"

Physiotherapists also commented on the feasibility of delivering the UK FROST physiotherapy programmes within the National Health Service (NHS) settings. There was a sense that it would be difficult to deliver the number of UK FROST physiotherapy sessions in routine practice.

Physiotherapist 4: "... *they* (Trial participants) *were seen with the 24 hours' post-surgery and they had twelve sessions which is a luxury because in our Trust, that is never, not going to happen and that never used to happen"*.

2) Expectations and preferences about UK FROST treatments

Although health professionals said that they maintained equipoise, some described mixed expectations regarding UK FROST treatments. For example, some felt that ESP would not be as effective as surgery.

Surgeon 7: "*My expectation is that physio won't work. My expectation is that the other two are probably Equivocal."*

Some felt that ACR would perform better than ESP, whilst others felt that MUA would be comparable to ACR. There were also those who expected similar outcomes across arms.

Physiotherapist 6*: "I'm expecting the MUAs to be surprisingly better than I would expect. I think the arthrolysis do great anyway and the physio is an unfair one."*

Surgeons and physiotherapists suggested hydro-dilatation would be an easy to use, less invasive and inexpensive treatment and an appropriate alternative to combat NHS surgery waiting lists. They felt hydro-dilatation should have been included as a potential comparator in UK FROST.

Surgeon 5: "*I'd definitely have a hydro-dilatation group because part of your trial is trying to work out if the cheaper operation is better than the more expensive operation and hydro-dilatations probably gained quite popularity since we started the trial design and it reflects current practice.*"

Physiotherapist 6: "*It needs hydro-dilatation in it. I personally think it gets really good results on a big bulk of trial participants and it's a wasted opportunity to have done this study and not have that as one of the arms".*

3) Factors that influence treatment outcomes

Similar to trial participants, surgeons and physiotherapists also felt that reduced pain, improved movement and function were important treatment outcomes. They considered that patient engagement and positive treatment expectations would contribute to better outcomes, whilst diabetes would have a negative impact on prognosis.

Physiotherapist 5: "*I think the expectations and belief is probably the most noticeable factor that affects people's outcome; if they believe something is the right thing, the best thing for them, they seem to do well.*"

Surgeon 8: *"...I've seen plenty of them of male diabetics that say, "Well, I've been stiff for three years," or two and a half years, that's not uncommon"*

4) Perceptions of trial participants' experience in the trial

Surgeons and physiotherapists described that some patients declined to take part in the UK FROST because they had previous physiotherapy that did not work and hence wanted to avoid randomisation into the ESP.

Surgeon 8: "*Many of them they say, "Look, I would love to contribute to the greater good and be involved in clinical trials, but I've come to the point that I will not consent for physiotherapy if I was randomised to that...*"

They also described that participants came with fixed ideas of what treatment they wanted in the trial.

For peer review only

Physiotherapist 8: "*So, we saw a lot of frozen shoulders coming in, but a lot of them had fixed ideas of what treatment they wanted. They didn't want surgery yet, or they didn't want to take time off work was the other one, but less so. The standout one was they didn't want an operation, or they wanted to try physiotherapy and injection and then they would opt for surgery. They wanted it to be continuum like that, not a one or the other*".

However, surgeons and physiotherapists felt that trial participants found all the treatment arms acceptable once they were in the trial.

Surgeon 6: "*Once they had consented to be part of the study, and they had no problems because they were equally welcome on what treatment they would get… all those who once we enrolled them on to the study, were okay with that.*"

Physiotherapists described how trial participants were surprised with the number of post-procedural physiotherapy sessions they received.

Physiotherapist 1: "*I think they've been grateful and surprised at the amount of treatment* (Post-procedural physiotherapy) *that they are allowed to have following the procedure.*"

DISCUSSION

Principal findings

We interviewed the trial participants and healthcare professionals (surgeons and physiotherapists) to understand their experiences and perceptions of participating in the UK FROST. Our key findings were: Trial participants reported improved treatment outcomes and satisfaction with the trial treatments. All those interviewed had their personal treatment preferences and expectations about UK FROST treatments. Exercise adherence was considered important for better treatment outcomes.

Pain relief and return of shoulder movements and function are important treatment outcomes

Trial participants and healthcare professionals agreed that pain relief and improvement in function and movement were important treatment outcomes. Their priorities are similar to the results of a previous survey in 225 health care professionals in the UK [17] and three systematic reviews on shoulder outcomes [18-20].

Adherence to prescribed exercises is important for better outcomes

Trial participants and healthcare professionals described continued patient engagement with the prescribed exercise as important for better outcomes [21-23]. During the supervised exercise phase in the trial, UK FROST participants were motivated by treatment benefits [24] and did their home exercises regularly. They were also self-determined [24] to persevere

For peer review only

through the pain associated with exercise. However, after the trial, participants did not continue exercising because they thought they achieved enough movement to allow adequate daily function. Therefore, after the trial, they prioritised continuing their daily functional activities and ignored the occasional pain and mild restrictions during certain end-range movements. These findings are in line with a previous study which conceptualised participants' views on 'ideal' (no symptoms at all) and 'adequate' (return to function with residual deficits) recovery from musculoskeletal complaints [25].

Trial participants and healthcare professionals had their personal preferences/expectations about UK FROST treatments

It is natural and inevitable that patients have their own treatment preferences. It is well known that personal preferences have a psychological therapeutic impact on treatment outcomes [26, 27]. If participants get their preferred treatment in a trial, they are more likely to stay motivated, comply better and respond well. Participants who do not receive their preferred treatment might lack motivation and become less engaged. Though our findings indicate that trial participants had mixed preferences before participation, all UK FROST treatments were well-received at the end.

Our interview findings also indicated mixed expectations and preferences among UK FROST surgeons and physiotherapists, mainly based on their clinical experience [28-30]. Expert surgical and physiotherapy experience require years of skill building, training and repeated practice. It would be highly unlikely if experts did not acquire personal expectations or preferences. Some might argue that this is integral to developing expertise. This has significant implications in trials as it has an important impact on equipoise. Our findings confirm that personal treatment expectations do exist among healthcare professionals, especially when treatment decisions are mainly expertise-based rather than evidence-based in treatment decision-making.

Surgeons and physiotherapists also perceived hydrodilatation as easy to administer, less invasive and a cost-effective treatment for frozen shoulder instead of surgery. These findings resonate with its growing popularity and usage in clinical practice [31], in spite of a lack of sufficient evidence on efficacy and safety [32, 33].

Strengths and limitations

Interviews were conducted by a researcher not involved in UK FROST and by using open-ended questions that allowed interviewees to express their opinions freely. The interview codes and themes were reviewed by an experienced qualitative researcher to ensure rigour of analysis and interpretation of data.

This study has some limitations. Interviews were conducted with trial participants and healthcare professionals who participated in the UK FROST. Therefore, the findings may not be transferable outside this context. Secondly, only two trial participants who did not receive

For peer review only

their allocated treatments took part. This could have influenced their predominantly positive experiences with the trial treatments. Thirdly, 93% of the interviews were conducted via telephone. Therefore, we are uncertain if interviewees would have expressed differently in face-to-face interviews. Lastly, those who did not participate in the interviews may have had different experiences.

Implications for clinical practice

1. All UK FROST treatments were perceived as acceptable, beneficial, and satisfactory. The benefits and anticipated risks of these treatments must be clearly communicated to patients during shared treatment decision-making.

Implications for future research

1. Trial participants and healthcare professionals have preferences for treatments. Future studies should aim to understand how these preferences may influence trial results.

2. Implementation of UK FROST physiotherapy programmes within different NHS settings may be challenging. Pilot implementation studies to identify scaling-up strategies would be useful.

3. More large-scale and high-quality randomised controlled trials to ensure clinical effectiveness and safety of hydrodilatation are required to guide evidence-based practice.

CONCLUSION

This qualitative study has provided a fuller understanding of the perspectives of UK FROST trial participants and healthcare professionals. Future trial designs would usefully consider including qualitative research as part of intervention development to ensure feasibility of delivering treatments within the NHS and to guide implementation beyond the trial. Evaluation of hydro-dilation compared to other treatment options is favoured by healthcare professionals. A prognostic tool to assist timely advice and treatment for people at risk of poorer outcomes would be helpful.

For peer review only

FUNDING

This qualitative work was funded as part of the UK FROST Trial by the National Institute for Health Research (NIHR) Health Technology Assessment programme (Project reference 13/26/01).

COMPETING INTERESTS

Cynthia Srikesavan, Francine Toye, Charalambos P. Charalambous declare no competing interests.

Lorna Goodchild, Stephen Brealey, Matthew Northgraves report receiving grant from NIHR Health Technology Assessment programme that funded the trial.

Sarah E Lamb reports grants from the NIHR Health Technology Assessment Programme during the conduct of the study and was a member of several HTA boards from 2010 to 2015 and the NIHR CTU Standing Advisory Committee 2012–2016.

Amar Rangan reports other grants from NIHR HTA, ORUK and H2020 during the conduct of this study; and is a member of the NIHR i4i Challenge Awards Committee (2019-current). South Tees Hospitals NHS Foundation Trust receives educational grant to the department from DePuy Synthes (J&J limited). Institution also receives payment from DePuy Synthes (J&J limited) for Professor Rangan as the co-ordinating Investigator for the GLOBAL ICON Stemless Shoulder System Post Market Clinical Follow up Study: CT 1401. These are outside and unrelated to the submitted work.

ACKNOWLEDGEMENTS

The authors thank the patient contributors who took part in developing the interview guide and patients and healthcare professionals participated in the interviews. This work was supported by the National Institute for Health Research (NIHR) Oxford Biomedical Research Centre (BRC). The UK FROST team acknowledges the support of the NIHR Clinical Research Network.

CONTRIBUTORS

Cynthia Srikesavan and Francine Toye led on the study and drafted the manuscript. Matthew Northgraves and Stephen Braeley contributed to recruitment. Lorna Goodchild and Sarah E Lamb provided expertise as physiotherapists. Amar Rangan and Charalambos P. Charalambous provided expertise as orthopaedic surgeons. All authors read, provided feedback and approved the final version.

DATA SHARING STATEMENT

All data relevant to the study are included in the article or uploaded as supplementary information. Data are available upon reasonable request.

For peer review only

OPEN ACCESS

This is an open access article distributed in accordance with the Creative Commons Attribution Non Commercial (CC BY-NC 4.0) license, which permits others to distribute, remix, adapt, build upon this work non-commercially, and license their derivative works on different terms, provided the original work is properly cited, appropriate credit is given, any changes made indicated, and the use is non-commercial. See: http:// creative commons.org/ licenses/ by- nc/ 4. 0/.

REFERENCES

1. Rangan A, Gibson J, Brownson P, et al. Frozen shoulder. *Shoulder & elbow*. 2015 Oct; 7(4):299-307.
2. Mezian K, Chang KV. Frozen Shoulder. In: *StatPearls*. Treasure Island (FL): StatPearls Publishing; 2020. Available from: https://www.ncbi.nlm.nih.gov/books/NBK482162/.
3. Rangan A, Hanchard N, McDaid C. What is the most effective treatment for frozen shoulder? *BMJ*. 2016 Aug 23; 354:i4162.
4. Nagy MT, MacFarlane RJ, Yousaf Khan MW. Suppl 3: The Frozen Shoulder: Myths and Realities. *The open orthopaedics journal*. 2013; 7:352.
5. Cavalieri E, Servadio A, Berardi A, Tofani M, Galeoto G. The Effectiveness of Physiotherapy in Idiopathic or Primary Frozen Shoulder: a Systematic Review and Meta-Analysis. *Muscles, Ligaments & Tendons Journal (MLTJ)*. 2020 Jan 1; 10(1).
6. Sun Y, Chen J, Li H, Jiang J, et al. Steroid injection and nonsteroidal anti-inflammatory agents for shoulder pain: a PRISMA systematic review and meta-analysis of randomized controlled trials. *Medicine*. 2015 Dec; 94(50).
7. Le HV, Lee SJ, Nazarian A, Rodriguez EK. Adhesive capsulitis of the shoulder: review of pathophysiology and current clinical interventions. *Shoulder & elbow*. 2017 Apr; 9(2):75-84.
8. Manske RC, Prohaska D. Clinical commentary and literature review: diagnosis, conservative and surgical management of adhesive capsulitis. *Shoulder & Elbow*. 2010 Oct; 2(4):238-54.
9. Sharma S, Jacobs L. Management of frozen shoulder–conservative vs surgical? *The Annals of The Royal College of Surgeons of England*. 2011 Jul; 93(5):343-343.
10. Brealey S, Armstrong AL, Brooksbank A, et al. United Kingdom Frozen Shoulder Trial (UK FROST), multi-centre, randomised, 12 month, parallel group, superiority study to compare the clinical and cost-effectiveness of Early Structured Physiotherapy versus manipulation under anaesthesia versus arthroscopic capsular release for trial participants referred to secondary care with a primary frozen shoulder: study protocol for a randomised controlled trial. *Trials*. 2017 Dec; 18(1):614.
11. Carter B. Clients' experiences of frozen shoulder and its treatment with Bowen technique. *Complement Ther Nurs Midwifery* 2002; 8:204–10.

For peer review only

12. Jones S, Hanchard N, Hamilton S, Rangan A. A qualitative study of patients' perceptions and priorities when living with primary frozen shoulder. *BMJ open*. 2013 Sep 1; 3(9):e003452.
13. Charmaz, K. (2006). Constructing Grounded Theory – A Practical Guide through Qualitative Analysis. SAGE.
14. Rindflesch AB. A grounded-theory investigation of patient education in physical therapy practice. *Physiotherapy theory and practice*. 2009 Jun; 25(3):193-202.
15. NVivo qualitative data analysis Software; QSR International Pty Ltd. Version 11, 2012.
16. O'Brien BC, Harris IB, Beckman TJ, Reed DA, Cook DA. Standards for reporting qualitative research: a synthesis of recommendations. *Academic Medicine*. 2014 Sep 1; 89(9):1245-51.
17. Rodgers S, Brealey S, Jefferson L, et al. Exploring the outcomes in studies of primary frozen shoulder: is there a need for a core outcome set? *Quality of Life Research*. 2014 Nov 1; 23(9):2495-504.
18. Maund E, Craig D, Suekarran S, et al. Management of frozen shoulder: a systematic review and cost-effectiveness analysis. *Health Technology Assessment* (Winchester, England). 2012; 16(11):1.
19. Page MJ, Huang H, Verhagen AP, et al. Outcome reporting in randomized trials for shoulder disorders: Literature review to inform the development of a core outcome set. *Arthritis care & research*. 2018 Feb; 70(2):252-9.
20. Page MJ, McKenzie JE, Green SE, et al. Core domain and outcome measurement sets for shoulder pain trials are needed: systematic review of physical therapy trials. *Journal of clinical epidemiology*. 2015 Nov 1; 68(11):1270-81.
21. Bailey DL, Holden MA, Foster NE, et al. Defining adherence to therapeutic exercise for musculoskeletal pain: a systematic review. *Br J Sports Med* 2018; 10.1136/bjsports-2017-098742. http://dx.doi.org/10.1136/bjsports-2017-098742.
22. Chester R, Jerosch-Herold C, Lewis J, et al. Psychological factors are associated with the outcome of physiotherapy for people with shoulder pain: a multicentre longitudinal cohort study. *Br J Sports Med* 2018;52:269-75. http://dx.doi.org/10.1136/bjsports-2016-096084.
23. Picorelli AM, Pereira LS, Pereira DS, et al. Adherence to exercise programs for older people is influenced by program characteristics and personal factors: a systematic review. *J Physiother* 2014;60:151-6. http://dx.doi.org/10.1016/j.jphys.2014.06.012.
24. Teixeira PJ, Carraca EV, Markland D, et al. Exercise, physical activity, and self-determination theory: a systematic review. *Int J Behav Nutr Phys Act* 2012;9:78. http://dx.doi.org/10.1186/1479-5868-9-78.
25. Carroll LJ, Lis A, Weiser S, Torti J. How Well Do You Expect to Recover, and What Does Recovery Mean, Anyway? Qualitative Study of Expectations After a Musculoskeletal Injury. *Phys Ther* 2016;96:797-807. http://dx.doi.org/10.2522/ptj.20150229.
26. Preference Collaborative Review Group. Patients' preferences within randomised trials: systematic review and patient level meta-analysis. *BMJ* 2008;337: a1864. http://dx.doi.org/10.1136/bmj.a1864.

For peer review only

27. Walter SD, Turner RM, Macaskill P, et al. Estimation of treatment preference effects in clinical trials when some participants are indifferent to treatment choice. *BMC Med Res Methodol* 2017;17:29. http://dx.doi.org/10.1186/s12874-017-0304-x.
28. Cook C, Sheets C. Clinical equipoise and personal equipoise: two necessary ingredients for reducing bias in manual therapy trials. *J Man Manip Ther* 2011;19:55-7. http://dx.doi.org/10.1179/106698111x12899036752014.
29. Ghogawala Z, Barker FG, Carter BS. Clinical equipoise and the surgical randomized controlled trial. *Neurosurgery*. 2008 Jun 1; 62(6):N9-10.
30. Kwaees TA, Charalambous CP. Surgical and non-surgical treatment of frozen shoulder. Survey on surgeons treatment preferences. *Muscles Ligaments Tendons J* 2014;4:420-4.
31. Heire P BR, Mubashar M, Bhatti W. Ultrasound-guided hydrodilatation for adhesive capsulitis–a step-by-step guide. European Congress of Radiology 2015.
32. Catapano M, Mittal N, Adamich J, Kumbhare D, Sangha H. Hydrodilatation with corticosteroid for the treatment of adhesive capsulitis: a systematic review. PM&R. 2018 Jun; 10(6):623-35.
33. Saltychev M, Laimi K, Virolainen P, Fredericson M. Effectiveness of Hydrodilatation in Adhesive Capsulitis of Shoulder: A Systematic Review and Meta-Analysis. Scand J Surg. 2018; 107(4):285-93.

FIGURE LEGENDS

Figure 1: Interviews with trial participants. ESP: Early Structured Physiotherapy; MUA: Manipulation Under Anaesthesia; ACR: Arthroscopic Capsular Release.

Figure 2: Interviews with surgeons and physiotherapists.

For peer review only

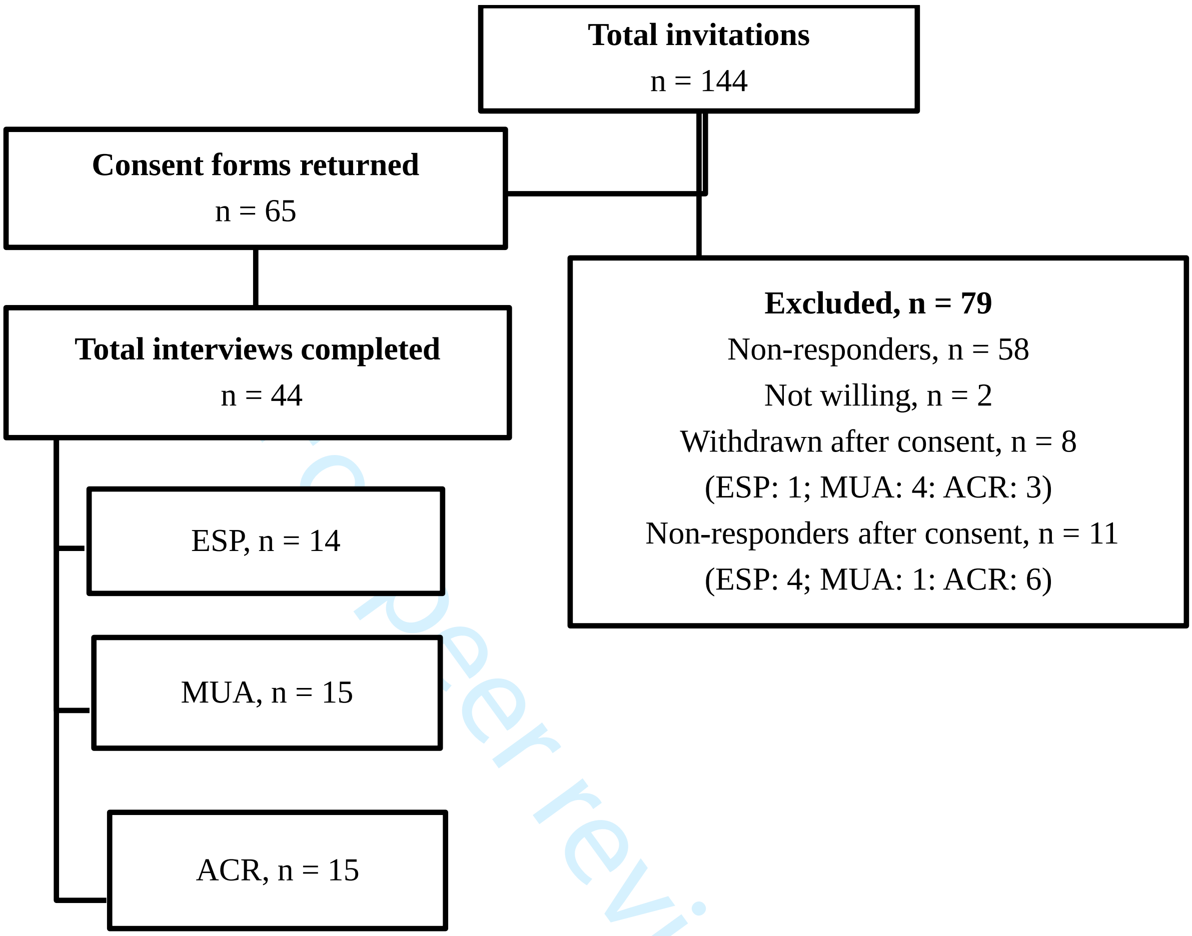

Figure 1: Interviews with trial participants. ESP: Early Structured Physiotherapy; MUA: Manipulation Under Anaesthesia; ACR: Arthroscopic Capsular Release.

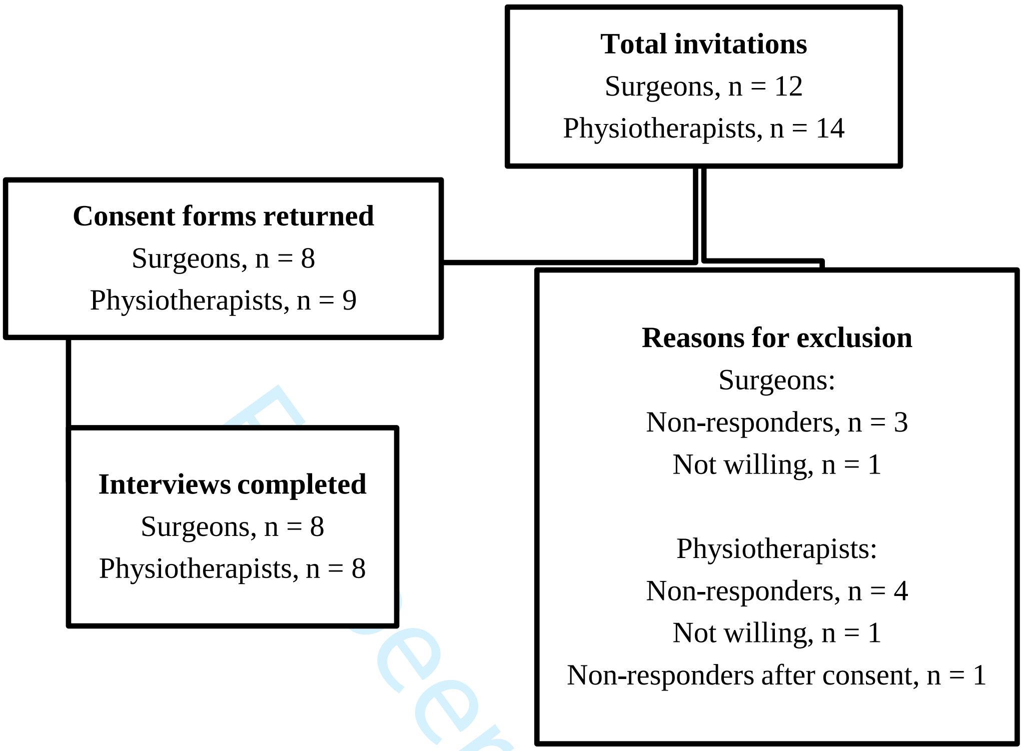

Figure 2: Interviews with surgeons and physiotherapists.

Reporting checklist for qualitative study.

Based on the SRQR guidelines.

Instructions to authors

Complete this checklist by entering the page numbers from your manuscript where readers will find each of the items listed below.

Your article may not currently address all the items on the checklist. Please modify your text to include the missing information. If you are certain that an item does not apply, please write "n/a" and provide a short explanation.

Upload your completed checklist as an extra file when you submit to a journal.

In your methods section, say that you used the SRQRreporting guidelines, and cite them as:

O'Brien BC, Harris IB, Beckman TJ, Reed DA, Cook DA. Standards for reporting qualitative research: a synthesis of recommendations. Acad Med. 2014;89(9):1245-1251.

		Reporting Item	Page Number
**Title**			
	#1	Concise description of the nature and topic of the study identifying the study as qualitative or indicating the approach (e.g. ethnography, grounded theory) or data collection methods (e.g. interview, focus group) is recommended	1
**Abstract**			
	#2	Summary of the key elements of the study using the abstract format of the intended publication; typically includes background, purpose, methods, results and conclusions	2
**Introduction**			
Problem formulation	#3	Description and significance of the problem / phenomenon studied: review of relevant theory and empirical work; problem statement	4
Purpose or research	#4	Purpose of the study and specific objectives or	4

For peer review only

question		questions	
**Methods**			
Qualitative approach and research paradigm	#5	Qualitative approach (e.g. ethnography, grounded theory, case study, phenomenolgy, narrative research) and guiding theory if appropriate; identifying the research paradigm (e.g. postpositivist, constructivist / interpretivist) is also recommended; rationale. The rationale should briefly discuss the justification for choosing that theory, approach, method or technique rather than other options available; the assumptions and limitations implicit in those choices and how those choices influence study conclusions and transferability. As appropriate the rationale for several items might be discussed together.	5
Researcher characteristics and reflexivity	#6	Researchers' characteristics that may influence the research, including personal attributes, qualifications / experience, relationship with participants, assumptions and / or presuppositions; potential or actual interaction between researchers' characteristics and the research questions, approach, methods, results and / or transferability	4
Context	#7	Setting / site and salient contextual factors; rationale	4
Sampling strategy	#8	How and why research participants, documents, or events were selected; criteria for deciding when no further sampling was necessary (e.g. sampling saturation); rationale	4, 5
Ethical issues pertaining to human subjects	#9	Documentation of approval by an appropriate ethics review board and participant consent, or explanation for lack thereof; other confidentiality and data security issues	4
Data collection methods	#10	Types of data collected; details of data collection procedures including (as appropriate) start and stop dates of data collection and analysis, iterative process, triangulation of sources / methods, and modification of procedures in response to evolving	4

For peer review only

		study findings; rationale	
Data collection instruments and technologies	#11	Description of instruments (e.g. interview guides, questionnaires) and devices (e.g. audio recorders) used for data collection; if / how the instruments(s) changed over the course of the study	4
Units of study	#12	Number and relevant characteristics of participants, documents, or events included in the study; level of participation (could be reported in results)	Figure 1 & 2
Data processing	#13	Methods for processing data prior to and during analysis, including transcription, data entry, data management and security, verification of data integrity, data coding, and anonymisation / deidentification of excerpts	4
Data analysis	#14	Process by which inferences, themes, etc. were identified and developed, including the researchers involved in data analysis; usually references a specific paradigm or approach; rationale	5
Techniques to enhance trustworthiness	#15	Techniques to enhance trustworthiness and credibility of data analysis (e.g. member checking, audit trail, triangulation); rationale	5
**Results/findings**			
Syntheses and interpretation	#16	Main findings (e.g. interpretations, inferences, and themes); might include development of a theory or model, or integration with prior research or theory	5-12
Links to empirical data	#17	Evidence (e.g. quotes, field notes, text excerpts, photographs) to substantiate analytic findings	5-12, Supplementary file
**Discussion**			
Intergration with prior work, implications, transferability and contribution(s) to the field	#18	Short summary of main findings; explanation of how findings and conclusions connect to, support, elaborate on, or challenge conclusions of earlier scholarship; discussion of scope of application / generalizability; identification of unique contributions(s) to scholarship in a discipline or field	13

For peer review only

Limitations	#19	Trustworthiness and limitations of findings	14
**Other**			
Conflicts of interest	#20	Potential sources of influence of perceived influence on study conduct and conclusions; how these were managed	16
Funding	#21	Sources of funding and other support; role of funders in data collection, interpretation and reporting	16

None The SRQR checklist is distributed with permission of Wolters Kluwer © 2014 by the Association of American Medical Colleges. This checklist can be completed online using https://www.goodreports.org/, a tool made by the EQUATOR Network in collaboration with Penelope.ai

For peer review only

BMJ Open

The experiences and perceptions of trial participants and healthcare professionals in the United Kingdom Frozen Shoulder Trial (UK FROST): A nested qualitative study.

Journal:	*BMJ Open*
Manuscript ID	bmjopen-2020-040829.R2
Article Type:	Original research
Date Submitted by the Author:	01-Apr-2021
Complete List of Authors:	Srikesavan, Cynthia; University of Oxford, Nuffield Department of Orthopaedics, Rheumatology, and Musculoskeletal Sciences<br>Toye, Francine; Oxford University Hospitals NHS Foundation Trust Nuffield Orthopaedic Centre, Physiotherapy Research Unit<br>Brealey, Stephen; University of York, York Trials Unit, ARRC Building, Department of Health Sciences.<br>Goodchild, Lorna; The Physiotherapy Practice<br>Northgraves, Matthew; Hull York Medical School<br>Charalambous, C; Blackpool Teaching Hospitals NHS Foundation Trust; University of Central Lancashire, School of Medicine<br>Rangan, Amar; University of York, York Trials Unit, ARRC Building, Department of Health Sciences<br>Lamb, Sarah; University of Exeter, College of Medicine and Health; University of Oxford, Nuffield Department of Orthopaedics, Rheumatology, and Musculoskeletal Sciences
**Primary Subject Heading**:	Qualitative research
Secondary Subject Heading:	Qualitative research
Keywords:	Elbow & shoulder < ORTHOPAEDIC & TRAUMA SURGERY, Musculoskeletal disorders < ORTHOPAEDIC & TRAUMA SURGERY, Shoulder < ORTHOPAEDIC & TRAUMA SURGERY

SCHOLARONE™
Manuscripts

BMJ

I, the Submitting Author has the right to grant and does grant on behalf of all authors of the Work (as defined in the below author licence), an exclusive licence and/or a non-exclusive licence for contributions from authors who are: i) UK Crown employees; ii) where BMJ has agreed a CC-BY licence shall apply, and/or iii) in accordance with the terms applicable for US Federal Government officers or employees acting as part of their official duties; on a worldwide, perpetual, irrevocable, royalty-free basis to BMJ Publishing Group Ltd ("BMJ") its licensees and where the relevant Journal is co-owned by BMJ to the co-owners of the Journal, to publish the Work in this journal and any other BMJ products and to exploit all rights, as set out in our licence.

The Submitting Author accepts and understands that any supply made under these terms is made by BMJ to the Submitting Author unless you are acting as an employee on behalf of your employer or a postgraduate student of an affiliated institution which is paying any applicable article publishing charge ("APC") for Open Access articles. Where the Submitting Author wishes to make the Work available on an Open Access basis (and intends to pay the relevant APC), the terms of reuse of such Open Access shall be governed by a Creative Commons licence – details of these licences and which Creative Commons licence will apply to this Work are set out in our licence referred to above.

Other than as permitted in any relevant BMJ Author's Self Archiving Policies, I confirm this Work has not been accepted for publication elsewhere, is not being considered for publication elsewhere and does not duplicate material already published. I confirm all authors consent to publication of this Work and authorise the granting of this licence.

For peer review only

Title page

Title of the article

The experiences and perceptions of trial participants and healthcare professionals in the United Kingdom Frozen Shoulder Trial (UK FROST): A nested qualitative study.

Full name, postal address and e-mail of the corresponding author

Dr Francine Toye (Ph.D, M.A Archaeology & Anthropology Cantab)
Qualitative Research Lead, Physiotherapy Research Unit,
Nuffield Orthopaedic Centre,
Oxford University Hospitals NHS Foundation Trust,
Windmill Road, Oxford OX3 7HE.
United Kingdom.

Email: francine.toye@ouh.nhs.uk.

Full name, department, institution, city and country of all co-authors
[1]Cynthia Srikesavan, [2]Francine Toye, [3]Stephen Brealey, [4]Lorna Goodchild, [5]Matthew Northgraves, [6]Charalambos P. Charalambous, [7]Amar Rangan, [8]Sarah E Lamb.

Affiliations
[1]Nuffield Department of Orthopaedics, Rheumatology, and Musculoskeletal Sciences, University of Oxford, Windmill road, Oxford, United Kingdom.
[2]Nuffield Orthopaedic Centre, Oxford University Hospitals NHS Foundation Trust, Windmill Road, Oxford, United Kingdom.
[3, 7]York Trials Unit, ARRC building, Department of Health Sciences, University of York, ARRC Building, York, United Kingdom.
[4]The Physiotherapy Practice, South Shields, United Kingdom.
[5]Hull York Medical School, Hull, United Kingdom.
[6]Blackpool Teaching Hospitals NHS Trust, Blackpool, and School of Medicine, University of Central Lancashire, Preston, United Kingdom.
[8]College of Medicine and Health, University of Exeter & Nuffield Department of Orthopaedics, Rheumatology, and Musculoskeletal Sciences, University of Oxford, Windmill road, Oxford, United Kingdom.

Word count, excluding title page, abstract, references, figures and tables: 4697

Keywords: Adhesive capsulitis; Arthroscopic capsular release; Frozen shoulder; Interviews; Manipulation under anaesthesia; Physiotherapy.

Abstract

Abstract word count: 298

Objectives

To explore the experiences and perceptions of trial participants and healthcare professionals in the United Kingdom Frozen Shoulder Trial (UK FROST), a multi-centre randomised controlled trial that compared manipulation under anaesthesia (MUA), arthroscopic capsular release (ACR) with a 12-week early structured physiotherapy programme (ESP) in people with unilateral frozen shoulder referred to secondary care.

Design

Nested qualitative study with semi-structured interviews. We used constant comparison method to develop our themes.

Setting

This qualitative study was nested within the UK FROST.

Participants

44 trial participants (ESP: 14; MUA: 15; ACR: 15), and 8 surgeons and 8 physiotherapists who delivered the treatments in the trial.

Results

Trial participants found UK FROST treatments acceptable and satisfactory in terms of content, delivery and treatment benefits. Participants in all arms experienced improvements in pain, shoulder movements, and function. Participants said they would choose the same treatment that they received in the trial.

Surgeons and physiotherapists felt that the content and delivery of UK FROST treatments was not significantly different to their routine practice except for the additional number of physiotherapy sessions offered in the trial. They had mixed feelings about the effectiveness of UK FROST treatments. Both stressed the value of including hydro-dilatation as a comparator of other treatment options. Physiotherapists raised concerns about the capacity to deliver the number of UK FROST physiotherapy sessions in routine clinical settings.

Shared perceptions of trial participants, surgeons and physiotherapists were: 1) Pain relief and return of shoulder movements and function are important outcomes; and 2) Adherence to exercises leads to better outcomes.

Conclusion

In general, our findings indicated that trial participants, and surgeons and physiotherapists who delivered the treatments had positive experiences and perceptions in the UK FROST. Early qualitative investigations to explore the feasibility of delivering treatments in real-world settings are suggested in future trials in frozen shoulder.

For peer review only

Keywords: Adhesive capsulitis; Arthroscopic capsular release; Frozen shoulder; Interviews; Manipulation under anaesthesia; Physiotherapy.

ARTICLE SUMMARY

Strengths and limitations of this study

- Interviews were conducted and analysed by a researcher who was not involved in the UK FROST.
- Interview codes and themes were reviewed by an experienced researcher to ensure rigour of analysis and interpretation of data.
- Interviews were conducted with participants and healthcare professionals who participated in the UK FROST. Therefore, the findings may not be transferable outside this context.
- 93% of the interviews were telephone-based. It is uncertain if interviewees would have expressed differently in face-to-face interviews.
- Those who did not participate in the interviews may have had different experiences.

For peer review only

Main text word count: 4227 (excluding Tables and Footnotes)

INTRODUCTION

Frozen shoulder or adhesive capsulitis is a chronic shoulder condition commonly affecting people aged between 50 and 60 years [1]. Women are affected four times than men [2], while the prevalence rate is around 20% in people with diabetes [2]. Frozen shoulder is characterised by three clinical stages that often overlap [3, 4]: 1) a painful stage (3 to 8 months), 2) a stiffness (frozen) stage (4 to 12 months) with progressive loss of active and passive shoulder movements and 3) a thawing stage (12 to 42 months) with gradual recovery of movements in most people [2].

Frozen shoulder is managed by non-surgical or surgical treatments [3, 5-9]. However, there is no high-quality evidence to recommend one treatment over another [3]. The United Kingdom Frozen Shoulder Trial (UK FROST) is a multi-centre, pragmatic randomised controlled trial in adults with frozen shoulder [10]. It evaluated the clinical and cost-effectiveness of two common surgical treatments: manipulation under anaesthesia (MUA) and arthroscopic capsular release (ACR) both followed by up to 12 weeks of post-procedural physiotherapy (PPP), with a 12-week early structured physiotherapy (ESP) programme that included an intra-articular steroid injection. Detailed descriptions of the trial treatments, including the specifically developed ESP and PPP pathways for the trial are available [10].

The qualitative literature on frozen shoulder is limited [11, 12]. There are no trials in frozen shoulder with an embedded qualitative component that explored the perspectives of trial participants (patients with frozen shoulder) and healthcare professionals who delivered the trial treatments. Therefore, we proposed a nested qualitative study within the UK FROST to explore the experiences and perceptions of trial participants who received UK FROST treatments, and healthcare professionals (surgeons and physiotherapists) who delivered those treatments.

METHODS

A qualitative and constructivist approach [13] was adopted to understand the experiences and perceptions of trial participants and healthcare professionals in the UK FROST.
The primary author (CS) is a physiotherapy researcher trained in qualitative methods and not involved in the delivery of UK FROST treatments and outcome measurement. CS conducted all the interviews and led the analysis. FT is an experienced qualitative researcher, anthropologist and physiotherapist, also not involved in delivery of care or outcome measurement. FT played a collaborative role in analysis.

Participants who took part in the main trial had primary unilateral frozen shoulder, identified through a restriction of passive external rotation on clinical examination. They were randomly assigned to MUA, ACR or ESP treatment arms in the ratio of 2:2:1. A purposive sample of trial participants was recruited from those who had agreed to be contacted by the

trial team for interviews at approximately 12 months post-randomisation, which coincided with the primary endpoint of the trial and completion of UK FROST treatments [10]. We proposed to recruit up to 45 trial participants. As gender and diabetes are likely to have an impact on the treatment outcomes [2, 10], we also planned to include an equal number of men and women, and those with and without diabetes.

We also planned to recruit a purposive sample of up to 15 surgeons and physiotherapists who delivered the treatments in the trial and agreed to take part in the interviews.

The study information sheet and consent form were posted to potential trial participants and emailed to surgeons and physiotherapists. Two postal or email reminders were sent to those who didn't respond within four weeks of invitation. Once signed consent was received via post or email from volunteer participants, surgeons and physiotherapists, CS contacted potential participants via telephone to coordinate an interview appointment with them.

Separate semi-structured interview guides with open-ended questions were used for trial participants and health professionals. Given the geographical spread of those participated, interviews were conducted face-to-face or via telephone. Interviews were audio-recorded using a digital recorder, anonymised and transcribed verbatim by a professional transcription agency.

The interviews were analysed using constant comparative method [13, 14] to develop categories and themes with a shared essence. The QSR International's NVivo 11 software [15] was used to organise and assist analysis of the data. The analysis was stopped at the point where additional data no longer contributed to the understanding of the themes. To enhance credibility of the findings, codes and themes were reviewed by an experienced qualitative researcher (FT). The standards for reporting qualitative research (SRQR) guidelines were used to report the study [16].

Patient and public involvement

The patient interview guide was developed following discussions with patients with frozen shoulder, research team, a physiotherapist and surgeon with expertise in this area, and reviewing the literature.

RESULTS

Sixty interviews (Participants: 44; Surgeons: 8 and Physiotherapists: 8) were completed between August 2016 and January 2018. Most interviews (56/60, 93%) were conducted via telephone. Four interviews were held at the clinic offices of surgeons and physiotherapists. Though constant comparison was used to analyse data, a formal theory was not developed.

A. Interviews with trial participants

For peer review only

A flow diagram showing the number of patients invited, excluded and participated in the interviews is presented in Figure 1. Participant characteristics are presented in Table 1. Apart from two participants, all other participants received their allocated treatment.

Five themes were identified. The themes were not different between men and women; and between those with and without diabetes.

Table 1: Characteristics of the participants interviewed

UK FROST arms	Male	Female	Age<br>Median (IQR) in years	Diabetic	Non-diabetic
ESP	5	9	58 (51 to 63.5)	3	11
MUA	8	7	55 (53 to 57.5)	5	10
ACR	7	8	59 (53 to 69)	5	10

UK FROST: United Kingdom Frozen Shoulder Trial; IQR: Inter-quartile range; ESP: Early Structured Physiotherapy; MUA: Manipulation Under Anaesthesia; ACR: Arthroscopic Capsular Release.

1) Experiences and perceptions of participating in the UK FROST

In general, trial participants had positive experiences in participating in UK FROST. They described altruistic and personal reasons for taking part in the trial. They were concerned about their upper limb disability from frozen shoulder and were keen on getting it sorted in UK FROST. There was also an overall sense that the trial questionnaires were relevant, simple and straightforward to complete.

> *"Because I'm all in favour, if you can do something to help other people not go through the misery that you've been through, and gone through, then I would do it".* (ESP)

Trial participants felt that their treatment packages were well coordinated and did not require any modification. The surgical procedures were clearly explained and physiotherapists who delivered ESP and PPP were supportive and helpful. Participants also liked to see the same physiotherapist for developing good connections and rapport throughout the study period. However, some ESP and ACR arm participants felt that the exercises were time consuming

For peer review only

and found it difficult to fit into their daily life. Some said that they would have liked more information about continuing exercises after the trial and exercising when in pain.

> *"Operation, I believe obviously went fantastic, went really well. It was just a case of waking up, recovery*."(ACR)

> *"Well personally I found it quite difficult to make sure I had them that many times in the day. I'm not too sure how somebody working or having a family could actually manage to fit it in because as I say, by the time going towards the middle to the end of the programme…"* (ESP)

2) Acceptability - Treatment satisfaction and improved outcomes

All UK FROST treatments were found to be acceptable, satisfactory and beneficial. Except for two participants in the ESP arm, others were satisfied with the UK FROST treatments they received. Of these two exceptions, one did not improve after physiotherapy in the trial and the other was not satisfied when the exercise sessions were supervised by an unfamiliar physiotherapist.

"I'm absolutely delighted with the treatment that I was given. I feel as though it did everything that I wanted it to do and expected it to do". (ESP)

"Very satisfied. I have no complaints at all" (ACR).

Participants considered pain relief and return of shoulder movements and function as important treatment outcomes. Participants in all arms experienced improvements in pain, movements and function. In spite of achieving pain relief and improved function, some participants said they experienced mild and occasional pain and restrictions during certain end-range activities. However, this did not impact their daily functioning.

Pain:
Trial participants experienced a substantial reduction in pain in all treatment arms. The ESP arm participants said that the steroid injections reduced pain and allowed them to start physiotherapy.

> *"When I went to the surgeon I was injected into my shoulder and the pain down my arm that more or less went straightaway."* (ESP)

> *"So at the beginning I said the pain was ten and now after all my physios, I'd say it was, I'd say it was about two now."* (MUA)

For peer review only

"I mean the pain in the beginning was just horrendous, it was really, really sore, really painful but after I'd had the physiotherapy, it was… I've got no pain at all now." (ACR)

Movements:

Trial participants described how the physiotherapy sessions (ESP and post-procedural) had helped to improve their shoulder movements.

"I could tell initially straightaway that my movement was starting to come, within a few days I could tell a difference of doing the exercises and as the weeks went on, it was just got better and better and by the time the twelve weeks was up, I virtually had full movements with no pain or anything, it was brilliant!" (ESP)

"After a few days I was doing my exercises and I was quite surprised already how much movement I had back and then it was regular physio appointments up at the hospital just to keep moving things around and that went really well…the physiotherapy was actually really, really beneficial." (MUA)

In the ACR arm, improvement in movements was thought to be quicker than participants had expected. Some experienced improvements as early as one to two weeks of physiotherapy after surgery.

"I felt that the physiotherapy I received was marvellous and improved the range of movements or showed me how to keep that range of movements much quicker than they did on the right-hand side, so I felt that everything went along fine, and I've got no complaints at all, none." (ACR)

"*It is almost like you have had a quick fix to fix your shoulder then you move on and I think personally for me because the surgery went very well and almost after a couple of weeks I was back to normal.*" (ACR)

Function:

Trial participants in all treatment arms described how their ability to do routine activities improved.

"I can lift my arm above my head now, you know? I can carry stuff, and I can lift it above my waist, and I can actually go swimming, you know? I can swim now." (ESP)

"My little everyday things have come back; I have come back, yes". (MUA)

"I can do everything – there's nothing that I can't do; I can wash my back, I can put my bra on, fasten it at the back, I can fasten my skirt at the side and the back now, there's nothing I can't do before I had the frozen shoulder everything I could do then I can now do again". (ACR)

For peer review only

3) Adherence to exercises

Trial participants described that ESP and PPP were difficult to begin with, but became easier on subsequent sessions.

> *"It* (Physiotherapy) *was agony but from every session there was slight improvement, so for every kind, every session I had, I had more movement and less pain, so it got to a point where some days I would go down and I had no pain at all."* (ESP)

However, participants were aware of the benefits of exercises and persevered with their home exercises during the study period.

> *"It was uncomfortable but I tried really hard with them to be honest because it was so awful when I couldn't use my arm that I knew that the only way to help it was to do them as much as I could."* (MUA)

Several participants said they did not continue their home exercises after the trial because they felt they had regained adequate shoulder function. Instead, they maintained their shoulder mobility by being functionally active. A few participants occasionally did some shoulder stretches.

> "*I'm working with my shoulder all the time so I'm not doing the exercises that the hospital gave me, because I'm working my, I'm swimming, I'm doing…I go on long walks, I take the dog out and what have you, so I'm using my arm.*" (ESP)

4) Personal treatment preferences

Trial participants had mixed treatment preferences before the trial. Some considered physiotherapy to be ineffective, while a few wanted to avoid the risks of surgery. Some preferred the less invasive MUA while some thought ACR as the final solution. Some didn't have any preference at all.

> *"I'm not really too sure why I wouldn't choose physiotherapy. I just think surgery seems a more final option. Physiotherapy, it might work, it may help, it may not. But to me, if I was given surgery, the surgery would work. I had more faith in the surgery working than the physiotherapy itself.*" (ACR)

> *"Well I didn't want to go to surgery or anything like that, so I just had the needle in my shoulder. I don't think I'd have wanted to go surgery at my age"* (ESP)

Despite preferences at the outset, at the end of the trial, there was a sense that participants would choose the same treatment they received in the UK FROST. In retrospect, some

For peer review only

participants in ESP and MUA said that they would choose surgery as a permanent and time-saving solution for their shoulder problem.

> *"Oh no I would have the same treatment. As I say I was only there for ten weeks and I mean, on my eleventh week I was still hell of a lot better."* (ESP*)*

B. Interviews with surgeons and physiotherapists

A flow diagram showing the number of health professionals invited, excluded and participated in the interviews is presented in Figure 2. The characteristics of surgeons and physiotherapists are presented in Table 2.

Four themes were identified.

Table 2: Characteristics of the surgeons and physiotherapists interviewed

	**Gender**		**Years of experience in treating shoulder conditions**
	Male	Female	Median (IQR) years
Surgeons	7	1	11 (7 to16.25)
Physiotherapists	--	8	13.5 (12 to 15.75)

IQR: Inter-quartile range

1) Experiences and perceptions of delivering UK FROST treatments

In general, surgeons and physiotherapists had positive experiences in delivering the trial treatments. Surgeons described how the surgical treatments were no different from their usual practice. They described how trial participants who received MUA or ACR were usually seen by specialised physiotherapists for advice and PPP at the follow-up stage. Some said there were no reports of complications or side-effects due to surgery, whilst some said that a few patients did not respond well to ESP.

> Surgeon 01: *"Technically there was no difference, I wouldn't do anything different".*

> Surgeon 06: *"In the physiotherapy group, there have been a couple of patients who have not responded very well to surgery, very well to the treatment, to the physiotherapy treatment and we have just carried on and they've taken a bit longer."*

Physiotherapists felt that the UK FROST physiotherapy programmes and the exercise booklet gave more flexibility in choosing the exercises than they would usually have in their routine practice. The only difference was the increased number of sessions offered in the trial. Surgical soreness was a commonly seen post-operative issue in ACR and MUA participants. However, it was quick to resolve with PPP compared to those who received ESP. There were a few suggestions from physiotherapists to improve the UK FROST exercise intervention. For example, one physiotherapist felt the exercises could have been standardised better. Two others suggested either spreading the 12 weekly sessions over 6 months or doing group sessions.

> Physiotherapist 1: "I *think the interventions that were on the booklet were what I would use generally. There was always an option there for me to tick off what I would do so I was in agreement with the options that were there and in agreement with the options that they actually, didn't want you to use*".

> Physiotherapist 5: "*I think group sessions would be really useful because patients get a lot from each other, and having experience group sessions with other clients with different pathologies, you know, they find that really reassuring…*"

Physiotherapists also commented on the feasibility of delivering the UK FROST physiotherapy programmes within the National Health Service (NHS) settings. There was a sense that it would be difficult to deliver the number of UK FROST physiotherapy sessions in routine practice.

> Physiotherapist 4: "*… they* (Trial participants) *were seen with the 24 hours' post-surgery and they had twelve sessions which is a luxury because in our Trust, that is never, not going to happen and that never used to happen".*

2) Expectations and preferences about UK FROST treatments

Although health professionals said that they maintained equipoise, some described mixed expectations regarding UK FROST treatments. For example, some felt that ESP would not be as effective as surgery.

> Surgeon 7: "*My expectation is that physio won't work. My expectation is that the other two are probably Equivocal."*

Some felt that ACR would perform better than ESP, whilst others felt that MUA would be comparable to ACR. There were also those who expected similar outcomes across arms.

> Physiotherapist 6*: "I'm expecting the MUAs to be surprisingly better than I would expect. I think the arthrolysis do great anyway and the physio is an unfair one."*

For peer review only

Surgeons and physiotherapists suggested hydro-dilatation would be an easy to use, less invasive and inexpensive treatment and an appropriate alternative to combat NHS surgery waiting lists. They felt hydro-dilatation should have been included as a potential comparator in UK FROST.

> Surgeon 5: "*I'd definitely have a hydro-dilatation group because part of your trial is trying to work out if the cheaper operation is better than the more expensive operation and hydro-dilatations probably gained quite popularity since we started the trial design and it reflects current practice.*"

> Physiotherapist 6: "*It needs hydro-dilatation in it. I personally think it gets really good results on a big bulk of trial participants and it's a wasted opportunity to have done this study and not have that as one of the arms".*

3) Factors that influence treatment outcomes

Similar to trial participants, surgeons and physiotherapists also felt that reduced pain, improved movement and function were important treatment outcomes. They considered that patient engagement and positive treatment expectations would contribute to better outcomes, whilst diabetes would have a negative impact on prognosis.

> Physiotherapist 5: "*I think the expectations and belief is probably the most noticeable factor that affects people's outcome; if they believe something is the right thing, the best thing for them, they seem to do well.*"

> Surgeon 8: *"...I've seen plenty of them of male diabetics that say, "Well, I've been stiff for three years," or two and a half years, that's not uncommon"*

4) Perceptions of trial participants' experience in the trial

Surgeons and physiotherapists described that some patients declined to take part in the UK FROST because they had previous physiotherapy that did not work and hence wanted to avoid randomisation into the ESP.

> Surgeon 8: "*Many of them they say, "Look, I would love to contribute to the greater good and be involved in clinical trials, but I've come to the point that I will not consent for physiotherapy if I was randomised to that...*"

They also described that participants came with fixed ideas of what treatment they wanted in the trial.

> Physiotherapist 8: "*So, we saw a lot of frozen shoulders coming in, but a lot of them had fixed ideas of what treatment they wanted. They didn't want surgery yet, or they didn't want to take time off work was the other one, but less so. The standout one was*

For peer review only

they didn't want an operation, or they wanted to try physiotherapy and injection and then they would opt for surgery. They wanted it to be continuum like that, not a one or the other".

However, surgeons and physiotherapists felt that trial participants found all the treatment arms acceptable once they were in the trial.

Surgeon 6: "*Once they had consented to be part of the study, and they had no problems because they were equally welcome on what treatment they would get… all those who once we enrolled them on to the study, were okay with that.*"

Physiotherapists described how trial participants were surprised with the number of post-procedural physiotherapy sessions they received.

Physiotherapist 1: "*I think they've been grateful and surprised at the amount of treatment* (Post-procedural physiotherapy) *that they are allowed to have following the procedure."*

DISCUSSION

Principal findings

We interviewed the trial participants and healthcare professionals (surgeons and physiotherapists) to understand their experiences and perceptions of participating in the UK FROST. Our key findings were: Trial participants reported improved treatment outcomes and satisfaction with the trial treatments. All those interviewed had their personal treatment preferences and expectations about UK FROST treatments. Exercise adherence was considered important for better treatment outcomes.

Pain relief and return of shoulder movements and function are important treatment outcomes

Trial participants and healthcare professionals agreed that pain relief and improvement in function and movement were important treatment outcomes. Their priorities are similar to the results of a previous survey in 225 health care professionals in the UK [17] and three systematic reviews on shoulder outcomes [18-20].

Adherence to prescribed exercises is important for better outcomes

Trial participants and healthcare professionals described continued patient engagement with the prescribed exercise as important for better outcomes [21-23]. During the supervised exercise phase in the trial, UK FROST participants were motivated by treatment benefits [24] and did their home exercises regularly. They were also self-determined [24] to persevere through the pain associated with exercise. However, after the trial, participants did not continue exercising because they thought they achieved enough movement to allow adequate daily function. Therefore, after the trial, they prioritised continuing their daily functional

For peer review only

activities and ignored the occasional pain and mild restrictions during certain end-range movements. These findings are in line with a previous study which conceptualised participants' views on 'ideal' (no symptoms at all) and 'adequate' (return to function with residual deficits) recovery from musculoskeletal complaints [25].

Trial participants and healthcare professionals had their personal preferences/expectations about UK FROST treatments

It is natural and inevitable that patients have their own treatment preferences. It is well known that personal preferences have a psychological therapeutic impact on treatment outcomes [26, 27]. If participants get their preferred treatment in a trial, they are more likely to stay motivated, comply better and respond well. Participants who do not receive their preferred treatment might lack motivation and become less engaged. Though our findings indicate that trial participants had mixed preferences before participation, all UK FROST treatments were well-received at the end.

Our interview findings also indicated mixed expectations and preferences among UK FROST surgeons and physiotherapists, mainly based on their clinical experience [28-30]. Expert surgical and physiotherapy experience require years of skill building, training and repeated practice. It would be highly unlikely if experts did not acquire personal expectations or preferences. Some might argue that this is integral to developing expertise. This has significant implications in trials as it has an important impact on equipoise. Our findings confirm that personal treatment expectations do exist among healthcare professionals, especially when treatment decisions are mainly expertise-based rather than evidence-based in treatment decision-making.

Surgeons and physiotherapists also perceived hydrodilatation as easy to administer, less invasive and a cost-effective treatment for frozen shoulder instead of surgery. These findings resonate with its growing popularity and usage in clinical practice [31], in spite of a lack of sufficient evidence on efficacy and safety [32, 33].

Strengths and limitations

Interviews were conducted by a researcher not involved in UK FROST and by using open-ended questions that allowed interviewees to express their opinions freely. The interview codes and themes were reviewed by an experienced qualitative researcher to ensure rigour of analysis and interpretation of data. We also discussed our preliminary findings with a team of physiotherapists who treat people with frozen shoulder and the UK FROST team to produce our final themes.'

This study has some limitations. Interviews were conducted with trial participants and healthcare professionals who participated in the UK FROST. Therefore, the findings may not be transferable outside this context. Secondly, only two trial participants who did not receive

For peer review only

their allocated treatments took part. This could have influenced their predominantly positive experiences with the trial treatments. Thirdly, 93% of the interviews were conducted via telephone. Therefore, we are uncertain if interviewees would have expressed differently in face-to-face interviews. Lastly, those who did not participate in the interviews may have had different experiences.

Implications for clinical practice

1. All UK FROST treatments were perceived as acceptable, beneficial, and satisfactory. The benefits and anticipated risks of these treatments must be clearly communicated to patients during shared treatment decision-making.

Implications for future research

1. Trial participants and healthcare professionals have preferences for treatments. Future studies should aim to understand how these preferences may influence trial results.

2. Implementation of UK FROST physiotherapy programmes within different NHS settings may be challenging. Pilot implementation studies to identify scaling-up strategies would be useful.

3. More large-scale and high-quality randomised controlled trials to ensure clinical effectiveness and safety of hydrodilatation are required to guide evidence-based practice.

CONCLUSION

This qualitative study has provided a fuller understanding of the perspectives of UK FROST trial participants and healthcare professionals. Future trial designs would usefully consider including qualitative research as part of intervention development to ensure feasibility of delivering treatments within the NHS and to guide implementation beyond the trial. Evaluation of hydro-dilation compared to other treatment options is favoured by healthcare professionals. A prognostic tool to assist timely advice and treatment for people at risk of poorer outcomes would be helpful.

For peer review only

FUNDING

This qualitative work was funded as part of the UK FROST Trial by the National Institute for Health Research (NIHR) Health Technology Assessment programme (Project reference 13/26/01).

COMPETING INTERESTS

Cynthia Srikesavan, Francine Toye, Charalambos P. Charalambous declare no competing interests.

Lorna Goodchild, Stephen Brealey, Matthew Northgraves report receiving grant from NIHR Health Technology Assessment programme that funded the trial.

Sarah E Lamb reports grants from the NIHR Health Technology Assessment Programme during the conduct of the study and was a member of several HTA boards from 2010 to 2015 and the NIHR CTU Standing Advisory Committee 2012–2016.

Amar Rangan reports other grants from NIHR HTA, ORUK and H2020 during the conduct of this study; and is a member of the NIHR i4i Challenge Awards Committee (2019-current). South Tees Hospitals NHS Foundation Trust receives educational grant to the department from DePuy Synthes (J&J limited). Institution also receives payment from DePuy Synthes (J&J limited) for Professor Rangan as the co-ordinating Investigator for the GLOBAL ICON Stemless Shoulder System Post Market Clinical Follow up Study: CT 1401. These are outside and unrelated to the submitted work.

ACKNOWLEDGEMENTS

The authors thank the patient contributors who took part in developing the interview guide and patients and healthcare professionals participated in the interviews. This work was supported by the National Institute for Health Research (NIHR) Oxford Biomedical Research Centre (BRC). The UK FROST team acknowledges the support of the NIHR Clinical Research Network.

CONTRIBUTORS

Cynthia Srikesavan and Francine Toye led on the study and drafted the manuscript. Matthew Northgraves and Stephen Braeley contributed to recruitment. Lorna Goodchild and Sarah E Lamb provided expertise as physiotherapists. Amar Rangan and Charalambos P. Charalambous provided expertise as orthopaedic surgeons. All authors read, provided feedback and approved the final version.

DATA SHARING STATEMENT

All data relevant to the study are included in the article or uploaded as supplementary information. Data are available upon reasonable request.

For peer review only

OPEN ACCESS

This is an open access article distributed in accordance with the Creative Commons Attribution Non Commercial (CC BY-NC 4.0) license, which permits others to distribute, remix, adapt, build upon this work non-commercially, and license their derivative works on different terms, provided the original work is properly cited, appropriate credit is given, any changes made indicated, and the use is non-commercial. See: http:// creative commons.org/ licenses/ by- nc/ 4. 0/.

ETHICS APPROVAL

Ethics approval was obtained from the NRES Committee North East -Newcastle and North Tyneside 2 ethics committee (14/NE/1176) and Health Research Authority (HRA).

REFERENCES

1. Rangan A, Gibson J, Brownson P, et al. Frozen shoulder. *Shoulder & elbow*. 2015 Oct; 7(4):299-307.
2. Mezian K, Chang KV. Frozen Shoulder. In: *StatPearls*. Treasure Island (FL): StatPearls Publishing; 2020. Available from: https://www.ncbi.nlm.nih.gov/books/NBK482162/.
3. Rangan A, Hanchard N, McDaid C. What is the most effective treatment for frozen shoulder? *BMJ*. 2016 Aug 23; 354:i4162.
4. Nagy MT, MacFarlane RJ, Yousaf Khan MW. Suppl 3: The Frozen Shoulder: Myths and Realities. *The open orthopaedics journal*. 2013; 7:352.
5. Cavalieri E, Servadio A, Berardi A, Tofani M, Galeoto G. The Effectiveness of Physiotherapy in Idiopathic or Primary Frozen Shoulder: a Systematic Review and Meta-Analysis. *Muscles, Ligaments & Tendons Journal (MLTJ)*. 2020 Jan 1; 10(1).
6. Sun Y, Chen J, Li H, Jiang J, et al. Steroid injection and nonsteroidal anti-inflammatory agents for shoulder pain: a PRISMA systematic review and meta-analysis of randomized controlled trials. *Medicine*. 2015 Dec; 94(50).
7. Le HV, Lee SJ, Nazarian A, Rodriguez EK. Adhesive capsulitis of the shoulder: review of pathophysiology and current clinical interventions. *Shoulder & elbow*. 2017 Apr; 9(2):75-84.
8. Manske RC, Prohaska D. Clinical commentary and literature review: diagnosis, conservative and surgical management of adhesive capsulitis. *Shoulder & Elbow*. 2010 Oct; 2(4):238-54.
9. Sharma S, Jacobs L. Management of frozen shoulder–conservative vs surgical? *The Annals of The Royal College of Surgeons of England*. 2011 Jul; 93(5):343-343.
10. Brealey S, Armstrong AL, Brooksbank A, et al. United Kingdom Frozen Shoulder Trial (UK FROST), multi-centre, randomised, 12 month, parallel group, superiority study to compare the clinical and cost-effectiveness of Early Structured Physiotherapy versus manipulation under anaesthesia versus arthroscopic capsular release for trial participants referred to secondary care with a primary frozen shoulder: study protocol for a randomised controlled trial. *Trials*. 2017 Dec; 18(1):614.

11. Carter B. Clients' experiences of frozen shoulder and its treatment with Bowen technique. *Complement Ther Nurs Midwifery* 2002; 8:204–10.
12. Jones S, Hanchard N, Hamilton S, Rangan A. A qualitative study of patients' perceptions and priorities when living with primary frozen shoulder. *BMJ open*. 2013 Sep 1; 3(9):e003452.
13. Charmaz, K. (2006). Constructing Grounded Theory – A Practical Guide through Qualitative Analysis. SAGE.
14. Rindflesch AB. A grounded-theory investigation of patient education in physical therapy practice. *Physiotherapy theory and practice*. 2009 Jun; 25(3):193-202.
15. NVivo qualitative data analysis Software; QSR International Pty Ltd. Version 11, 2012.
16. O'Brien BC, Harris IB, Beckman TJ, Reed DA, Cook DA. Standards for reporting qualitative research: a synthesis of recommendations. *Academic Medicine*. 2014 Sep 1; 89(9):1245-51.
17. Rodgers S, Brealey S, Jefferson L, et al. Exploring the outcomes in studies of primary frozen shoulder: is there a need for a core outcome set? *Quality of Life Research.* 2014 Nov 1; 23(9):2495-504.
18. Maund E, Craig D, Suekarran S, et al. Management of frozen shoulder: a systematic review and cost-effectiveness analysis. *Health Technology Assessment* (Winchester, England). 2012; 16(11):1.
19. Page MJ, Huang H, Verhagen AP, et al. Outcome reporting in randomized trials for shoulder disorders: Literature review to inform the development of a core outcome set. *Arthritis care & research.* 2018 Feb; 70(2):252-9.
20. Page MJ, McKenzie JE, Green SE, et al. Core domain and outcome measurement sets for shoulder pain trials are needed: systematic review of physical therapy trials. *Journal of clinical epidemiology*. 2015 Nov 1; 68(11):1270-81.
21. Bailey DL, Holden MA, Foster NE, et al. Defining adherence to therapeutic exercise for musculoskeletal pain: a systematic review. *Br J Sports Med* 2018; 10.1136/bjsports-2017-098742. http://dx.doi.org/10.1136/bjsports-2017-098742.
22. Chester R, Jerosch-Herold C, Lewis J, et al. Psychological factors are associated with the outcome of physiotherapy for people with shoulder pain: a multicentre longitudinal cohort study. *Br J Sports Med* 2018;52:269-75. http://dx.doi.org/10.1136/bjsports-2016-096084.
23. Picorelli AM, Pereira LS, Pereira DS, et al. Adherence to exercise programs for older people is influenced by program characteristics and personal factors: a systematic review. *J Physiother* 2014;60:151-6. http://dx.doi.org/10.1016/j.jphys.2014.06.012.
24. Teixeira PJ, Carraca EV, Markland D, et al. Exercise, physical activity, and self-determination theory: a systematic review. *Int J Behav Nutr Phys Act* 2012;9:78. http://dx.doi.org/10.1186/1479-5868-9-78.
25. Carroll LJ, Lis A, Weiser S, Torti J. How Well Do You Expect to Recover, and What Does Recovery Mean, Anyway? Qualitative Study of Expectations After a Musculoskeletal Injury. *Phys Ther* 2016;96:797-807. http://dx.doi.org/10.2522/ptj.20150229.

26. Preference Collaborative Review Group. Patients' preferences within randomised trials: systematic review and patient level meta-analysis. *BMJ* 2008;337: a1864. http://dx.doi.org/10.1136/bmj.a1864.
27. Walter SD, Turner RM, Macaskill P, et al. Estimation of treatment preference effects in clinical trials when some participants are indifferent to treatment choice. *BMC Med Res Methodol* 2017;17:29. http://dx.doi.org/10.1186/s12874-017-0304-x.
28. Cook C, Sheets C. Clinical equipoise and personal equipoise: two necessary ingredients for reducing bias in manual therapy trials. *J Man Manip Ther* 2011;19:55-7. http://dx.doi.org/10.1179/106698111x12899036752014.
29. Ghogawala Z, Barker FG, Carter BS. Clinical equipoise and the surgical randomized controlled trial. *Neurosurgery*. 2008 Jun 1; 62(6):N9-10.
30. Kwaees TA, Charalambous CP. Surgical and non-surgical treatment of frozen shoulder. Survey on surgeons treatment preferences. *Muscles Ligaments Tendons J* 2014;4:420-4.
31. Heire P BR, Mubashar M, Bhatti W. Ultrasound-guided hydrodilatation for adhesive capsulitis–a step-by-step guide. European Congress of Radiology 2015.
32. Catapano M, Mittal N, Adamich J, Kumbhare D, Sangha H. Hydrodilatation with corticosteroid for the treatment of adhesive capsulitis: a systematic review. PM&R. 2018 Jun; 10(6):623-35.
33. Saltychev M, Laimi K, Virolainen P, Fredericson M. Effectiveness of Hydrodilatation in Adhesive Capsulitis of Shoulder: A Systematic Review and Meta-Analysis. Scand J Surg. 2018; 107(4):285-93.

FIGURE LEGENDS

Figure 1: Interviews with trial participants. ESP: Early Structured Physiotherapy; MUA: Manipulation Under Anaesthesia; ACR: Arthroscopic Capsular Release.

Figure 2: Interviews with surgeons and physiotherapists.

For peer review only

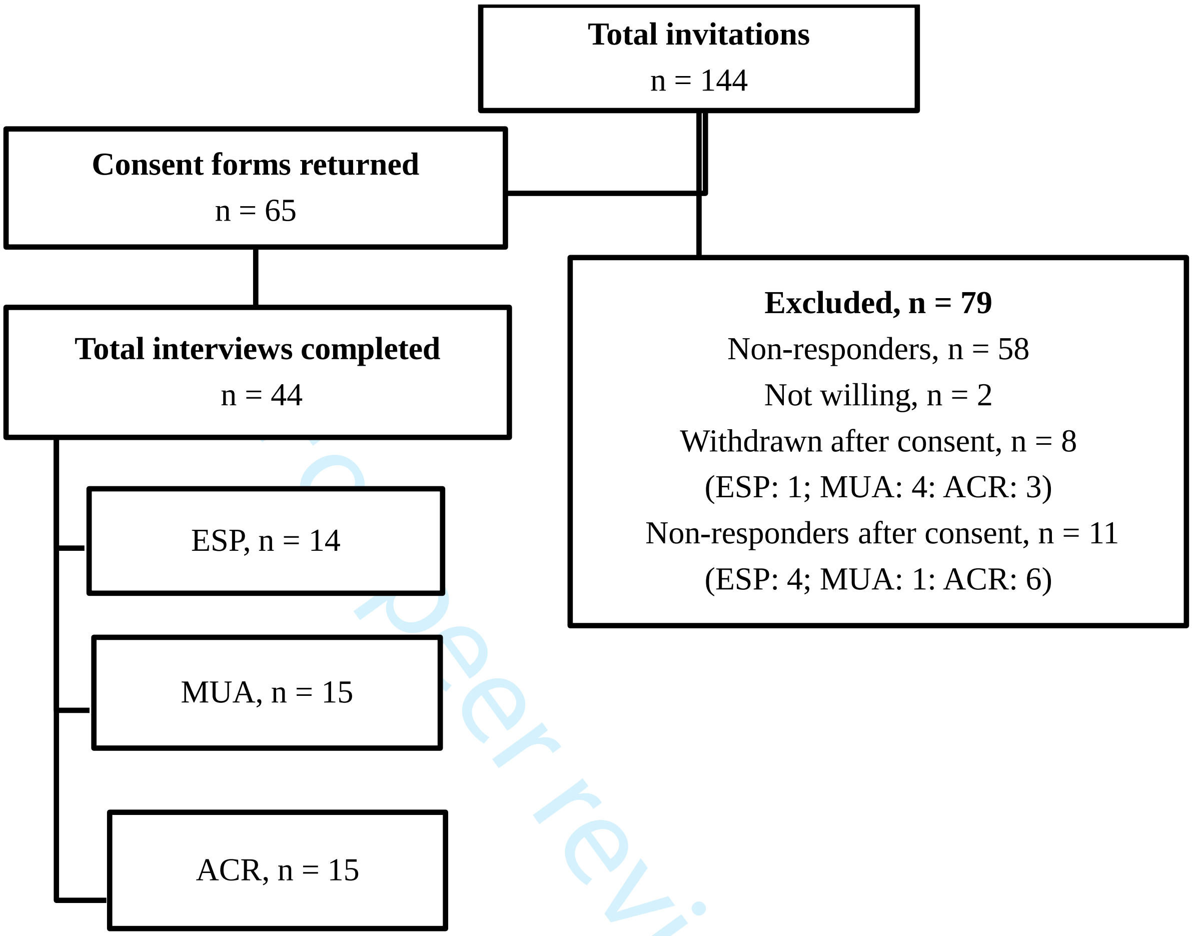

Figure 1: Interviews with trial participants. ESP: Early Structured Physiotherapy; MUA: Manipulation Under Anaesthesia; ACR: Arthroscopic Capsular Release.

For peer review only

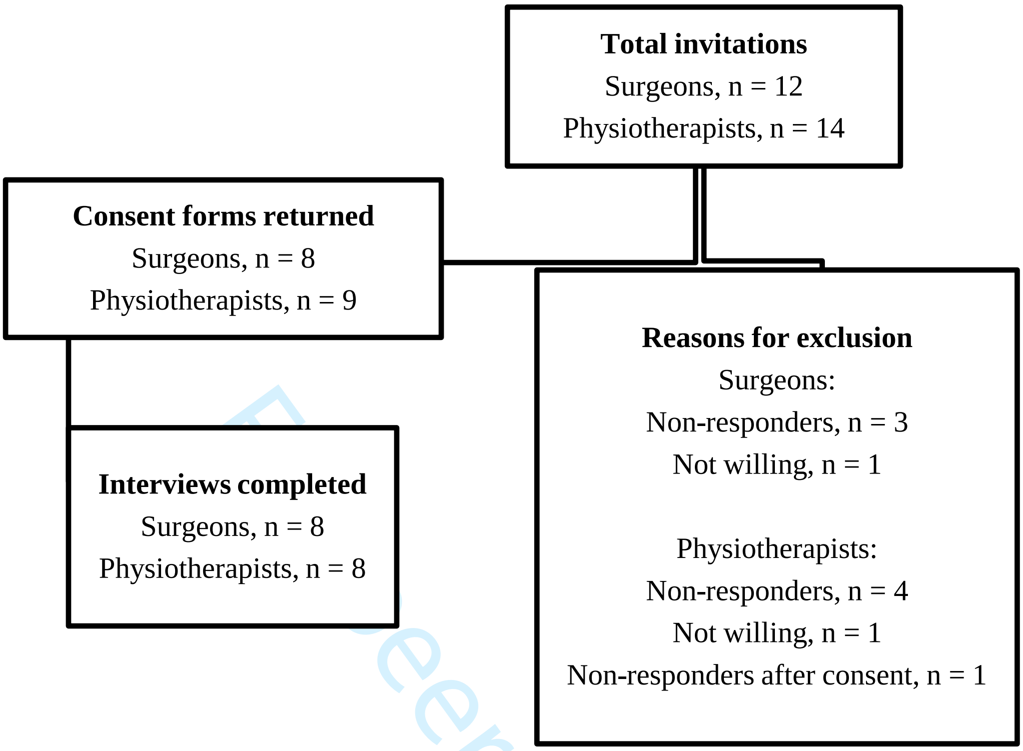

Figure 2: Interviews with surgeons and physiotherapists.

Reporting checklist for qualitative study.

Based on the SRQR guidelines.

Instructions to authors

Complete this checklist by entering the page numbers from your manuscript where readers will find each of the items listed below.

Your article may not currently address all the items on the checklist. Please modify your text to include the missing information. If you are certain that an item does not apply, please write "n/a" and provide a short explanation.

Upload your completed checklist as an extra file when you submit to a journal.

In your methods section, say that you used the SRQRreporting guidelines, and cite them as:

O'Brien BC, Harris IB, Beckman TJ, Reed DA, Cook DA. Standards for reporting qualitative research: a synthesis of recommendations. Acad Med. 2014;89(9):1245-1251.

		Reporting Item	Page Number
**Title**			
	#1	Concise description of the nature and topic of the study identifying the study as qualitative or indicating the approach (e.g. ethnography, grounded theory) or data collection methods (e.g. interview, focus group) is recommended	1
**Abstract**			
	#2	Summary of the key elements of the study using the abstract format of the intended publication; typically includes background, purpose, methods, results and conclusions	2
**Introduction**			
Problem formulation	#3	Description and significance of the problem / phenomenon studied: review of relevant theory and empirical work; problem statement	4
Purpose or research	#4	Purpose of the study and specific objectives or	4

For peer review only

question		questions	
**Methods**			
Qualitative approach and research paradigm	#5	Qualitative approach (e.g. ethnography, grounded theory, case study, phenomenolgy, narrative research) and guiding theory if appropriate; identifying the research paradigm (e.g. postpositivist, constructivist / interpretivist) is also recommended; rationale. The rationale should briefly discuss the justification for choosing that theory, approach, method or technique rather than other options available; the assumptions and limitations implicit in those choices and how those choices influence study conclusions and transferability. As appropriate the rationale for several items might be discussed together.	5
Researcher characteristics and reflexivity	#6	Researchers' characteristics that may influence the research, including personal attributes, qualifications / experience, relationship with participants, assumptions and / or presuppositions; potential or actual interaction between researchers' characteristics and the research questions, approach, methods, results and / or transferability	4
Context	#7	Setting / site and salient contextual factors; rationale	4
Sampling strategy	#8	How and why research participants, documents, or events were selected; criteria for deciding when no further sampling was necessary (e.g. sampling saturation); rationale	4, 5
Ethical issues pertaining to human subjects	#9	Documentation of approval by an appropriate ethics review board and participant consent, or explanation for lack thereof; other confidentiality and data security issues	4
Data collection methods	#10	Types of data collected; details of data collection procedures including (as appropriate) start and stop dates of data collection and analysis, iterative process, triangulation of sources / methods, and modification of procedures in response to evolving	4

For peer review only

		study findings; rationale	
Data collection instruments and technologies	#11	Description of instruments (e.g. interview guides, questionnaires) and devices (e.g. audio recorders) used for data collection; if / how the instruments(s) changed over the course of the study	4
Units of study	#12	Number and relevant characteristics of participants, documents, or events included in the study; level of participation (could be reported in results)	Figure 1 & 2
Data processing	#13	Methods for processing data prior to and during analysis, including transcription, data entry, data management and security, verification of data integrity, data coding, and anonymisation / deidentification of excerpts	4
Data analysis	#14	Process by which inferences, themes, etc. were identified and developed, including the researchers involved in data analysis; usually references a specific paradigm or approach; rationale	5
Techniques to enhance trustworthiness	#15	Techniques to enhance trustworthiness and credibility of data analysis (e.g. member checking, audit trail, triangulation); rationale	5
**Results/findings**			
Syntheses and interpretation	#16	Main findings (e.g. interpretations, inferences, and themes); might include development of a theory or model, or integration with prior research or theory	5-12
Links to empirical data	#17	Evidence (e.g. quotes, field notes, text excerpts, photographs) to substantiate analytic findings	5-12, Supplementary file
**Discussion**			
Intergration with prior work, implications, transferability and contribution(s) to the field	#18	Short summary of main findings; explanation of how findings and conclusions connect to, support, elaborate on, or challenge conclusions of earlier scholarship; discussion of scope of application / generalizability; identification of unique contributions(s) to scholarship in a discipline or field	13

For peer review only

Limitations	#19	Trustworthiness and limitations of findings	14
**Other**			
Conflicts of interest	#20	Potential sources of influence of perceived influence on study conduct and conclusions; how these were managed	16
Funding	#21	Sources of funding and other support; role of funders in data collection, interpretation and reporting	16

None The SRQR checklist is distributed with permission of Wolters Kluwer © 2014 by the Association of American Medical Colleges. This checklist can be completed online using https://www.goodreports.org/, a tool made by the EQUATOR Network in collaboration with Penelope.ai

For peer review only
